# An early relapse prediction model based on pathological features following neoadjuvant immunotherapy for hepatocellular carcinoma

**DOI:** 10.1093/oncolo/oyaf368

**Published:** 2025-11-10

**Authors:** Yanrui Pang, Xuelian Zhao, Xinxin Guo, Jing Han, Yuan Ji

**Affiliations:** Department of Pathology, Zhongshan Hospital, Fudan University, Shanghai, China; Department of Pathology, Shanghai Sixth People’s Hospital, Shanghai Jiao Tong University School of Medicine, Shanghai, China; Department of Pathology, Zhongshan Hospital, Fudan University, Shanghai, China; Department of Pathology, Zhongshan Hospital, Fudan University, Shanghai, China; Department of Pathology, Zhongshan Hospital, Fudan University, Shanghai, China; Department of Pathology, Zhongshan Hospital, Fudan University, Shanghai, China

**Keywords:** hepatocellular carcinoma, neoadjuvant, immunotherapy, recurrence, nomogram

## Abstract

**Background:**

The emergence of neoadjuvant immunotherapy has improved outcomes for hepatocellular carcinoma (HCC) patients, yet early postoperative recurrence remains a critical challenge. This study aimed to identify clinicopathological and immune microenvironment features associated with recurrence-free survival (RFS) and develop a predictive model for early recurrence in HCC patients undergoing neoadjuvant anti-PD-1 antibody therapy.

**Materials and Methods:**

Clinicopathological characteristics and immune microenvironment profiles were analyzed in 70 HCC patients treated with neoadjuvant anti-PD-1 antibody and 20 patients receiving transarterial chemoembolization (TACE). Key variables, including microvascular invasion (MVI), tumor capsule integrity, and immune cell infiltration, were evaluated. Statistical analyses included multivariate Cox regression, Kaplan-Meier survival analysis, and nomogram construction with internal validation to predict recurrence risk.

**Results:**

Foam cell response and tumor-infiltrating lymphocytes (TILs) were strongly linked to favorable immunotherapy responses. Patients without MVI, those with intact tumor capsules, and those exhibiting high CD4^+^ T cell density in central tumor regions demonstrated significantly prolonged RFS. A nomogram integrating these three factors achieved robust predictive accuracy for early recurrence stratifying patients into distinct risk groups.

**Conclusions:**

This study highlights MVI absence, tumor capsule integrity, and CD4^+^ T cell infiltration as key predictors of RFS in HCC patients receiving neoadjuvant immunotherapy. The proposed nomogram provides a clinically actionable tool for early recurrence risk assessment, enabling personalized postoperative monitoring and adjuvant therapy strategies to improve survival outcomes.

Implications for Practice This study presents a predictive nomogram model to assess early recurrence risk in HCC patients receiving neoadjuvant immunotherapy. Key clinicopathological and immune microenvironment factors, including the absence of MVI, intact tumor capsules, and high CD4^+^ T cell density, were identified as significant predictors of prolonged RFS. Clinically, this model can guide personalized postoperative monitoring and adjuvant treatment strategies, helping clinicians stratify patients based on recurrence risk. By integrating these predictive factors, the model supports improved decision-making, potentially leading to better survival outcomes through timely interventions.

## Introduction

In recent years, compelling evidence has emerged regarding the effectiveness of immunotherapy in treating various solid tumors, particularly through the use of immune checkpoint inhibitors (ICIs).^1–4^ Importantly, several studies have demonstrated promising results from combining immunotherapy with tyrosine kinase inhibitors (TKIs) for advanced hepatocellular carcinoma (HCC) patients.^5^ This combination therapy has shown potential in improving survival rates and delaying disease progression by simultaneously targeting multiple oncogenic pathways and modulating the immune microenvironment. However, despite these advancements, a significant proportion of HCC patients continue to experience early recurrence after receiving neoadjuvant immunotherapy.^6^

Early recurrence after neoadjuvant treatment poses a major obstacle to improving long-term survival outcomes in HCC. The underlying mechanisms of such recurrence are not yet fully understood, but recent studies suggest that both the pathological response of the tumor and the immune microenvironment play pivotal roles. For instance, the extent of tumor-infiltrating lymphocytes (TILs),^7^ the presence of tertiary lymphoid structures (TLSs),^8^^,^^9^ and other histopathological features may correlate with clinical outcomes and recurrence risk. Additionally, factors such as microvascular invasion (MVI)^10^^,^^11^ has been identified as potential indicators of poor prognosis following surgery. Therefore, understanding these key histopathological and immunological parameters is essential for predicting early recurrence and tailoring personalized treatment strategies.

To address this issue, we conducted a comprehensive study analyzing postoperative samples from HCC patients treated with neoadjuvant immunotherapy. The primary goal was to evaluate the pathological response, including the presence of viable tumor cells and immune-related histopathological features, such as TILs and foam cell response. Furthermore, we aimed to explore the immune microenvironment in these patients by assessing immune cell infiltration, particularly CD4^+^ T cell density, in various tumor regions. By integrating clinical, pathological, and immune factors, we sought to develop a predictive model that could better stratify patients by their risk of early postoperative recurrence. This model could provide valuable insights into improving postoperative management and guiding future therapeutic decisions for HCC patients undergoing neoadjuvant immunotherapy.

## Materials and methods

### Research subjects

This study enrolled a total of 90 patients with HCC who underwent surgical resection or liver transplantation at Zhongshan Hospital, Fudan University between 2019 and 2021. Among them, 70 patients were treated with anti-PD-1 Ab either alone or in combination with other therapies (TACE, TKIs) prior to surgery. Treatment-related data of these 70 patients, including administration time and dosage, are provided in Supplemental Material 1. In order to reduce the impact of other therapies on pathological remission, we have also selected 20 patients who underwent transcatheter arterial chemoembolization (TACE) alone before surgery. We also collected complete clinical and imaging data for each patient, including sex, age, viral infection status, antiviral treatment, the presence of liver cirrhosis, surgical approaches, and neoadjuvant therapy methods, etc This study was approved by the Research Ethics Committee of Zhongshan Hospital (B2022-155R), and informed consent was obtained from each patient.

### Sample processing

To provide a more comprehensive and visually accessible assessment of tumor regression in HCC patients following neoadjuvant therapy, we implemented extensive sampling of surgical resection specimens. Lesions with a maximum diameter of ≤ 3 cm should be entirely sampled. For lesions with a maximum diameter of > 3 cm, we cut the specimen at 1 cm interval, The characteristics of the tumor bed on each cut surface were visually inspected, and sampling was carried out on the cut surfaces with different regions. This approach not only ensures a comprehensive evaluation of the tumor regression bed but also significantly reduces the difficulty of complete sampling for massive HCC. For multiple lesions, we adopt the same sampling method for pathological evaluation based on the tumor size.

### Pathological assessment

Histopathological analysis: We examined various characteristics, including necrosis, fibrosis, bleeding, foam cell response, cholesterol crystallization, TILs, and TLSs. Among them, necrosis, fibrosis, bleeding, and foam cell response are scored based on their proportional representation within the overall tumor bed, the presence of these histological features is defined as ≥ 10%. Cholesterol crystallization, a common crystal-like structure in histological slices, signifies insoluble membrane lipid deposits often associated with necrosis. Due to the challenge in assessing regional proportions, the presence or absence of cholesterol crystallization is categorized based on its occurrence. Moreover, TILs are lymphocytes or tissue cells dispersed within the tumor cell nests, without forming specific structures. In this study, the existence of the TILs phenomenon is defined by the presence of inflammatory cells ≥ 10% within the tumor stroma.^12^ TLSs are aggregations of immune cells with a node-like structure.^13^ The presence or absence of TLSs is categorized based on its occurrence.

Residual tumor ratio: We assess the proportion of the residual viable tumor within the total tumor bed through all pathological hematoxylin and eosin (HE) slides. No viable tumor cells remain were defined as pathological complete response (pCR), tumors with low residual tumor ratio were defined as major pathological response (mPR).

### Immunohistochemistry (IHC)

Histopathological analysis: Pathological samples from 70 HCC tissue specimens were fixed in 4% neutral formaldehyde solution at room temperature for 24 hours, then embedded in paraffin and cut into 4-μm sections. These sections were deparaffinized with xylene (15 min; Shanghai Macklin Biochemical Co., Ltd) and hydrated through a series of alcohol solutions (100% ethanol I for 5 min; 100% ethanol II for 5 min; 95% ethanol for 3 min; 90% ethanol for 3 min; 80% ethanol for 2 min; and 70% ethanol for 2 min) (Shanghai Macklin Biochemical Co., Ltd). Antigen retrieval was performed using 10 mM citrate buffer (pH 6; Beijing Jimei Biotechnology Co., Ltd) at 100 °C for 10—15 minutes, and endogenous peroxidase activity was blocked with 3% hydrogen peroxide at 37 °C for 10 minutes. The sections were then incubated with specific antibodies overnight at 4 °C in a humidified chamber. The following antibodies used were: CD3 (clone LN10, dilution 1/500; Leica), CD4 (clone EP204, dilution 1/300; Gene Tech), CD8 (clone 4B11, dilution 1/300; Leica), FOXP3 (clone 236A/E7, dilution 1/300; Abcam), CD20 (clone L26, dilution 1/1000; DAKO), CD38 (clone 38C03, dilution 1/300; Gene Tech), CD68 (clone PGM1, dilution 1/300; DAKO), CD206 (dilution 1/6000; Abcam), PD-1 (clone EPR4877, dilution 1/1000; Abcam), PD-L1{28-8} (dilution 1/1000; Abcam), and PD-L1{22C3} (dilution 1/50; DAKO). The MaxVision kit (Fuzhou Maixin Biotechnology Development Co., Ltd) was used to detect primary antibodies. The color was developed with 3,3′-diaminobenzidine chromogen substrate for 10 minutes at room temperature and counterstained with hematoxylin at room temperature for 1 minute. Finally, the sections were dehydrated in graded ethanol, cleared, and mounted.

IHC positive cell count: Based on HE staining, the assessed regions include invasive margin (IM), central tumor (CT), and normal liver (NL). IHC slides were scanned using a digital pathological scanner. Positive cell counting was conducted in positive cell hotspots within these three regions. For the CT region, cell counting focuses on the viable tumor. For each region, 5 hotspot images were captured at 20x magnification, and the positive cell count per square millimeter was calculated using ImageJ software.^14^ Moreover, we categorized immune cells based on the median count of positive cells and then divided immune cells in different regions into high and low infiltration groups.

### Follow-up

A total of 90 patients were followed up using the electronic medical records system and telephone communication. Comprehensive records regarding patient survival, recurrence, and metastasis were meticulously maintained. Recurrence-free survival (RFS) measures the time from the initiation of surgery end to recurrence. OS gauges the period from treatment start to death or last follow-up.

### Statistical analysis

To examine the correlation between variables among different groups, we utilized the chi-square test. To assess the impact of clinicopathological and immune factors on the RFS of HCC patients, we performed Cox univariate and multivariate analyses. To construct a predictive model, we employed the nomogram component of R software. Additionally, we utilized receiver operating characteristic (ROC) curve analysis, decision curve analysis (DCA), integrated discrimination improvement (IDI), and net reclassification improvement (NRI) to calibrate and validate the model. We conducted statistical analysis using IBM SPSS 26.0 and GraphPad Prism 9 software. Statistical significance was determined based on two-sided *P* values, with a threshold of < 0.05.

## Results

### Clinicopathological characteristics of 90 HCC patients

A total of 90 HCC patients were included in this study. Among them, 16 (17.8%) patients only received anti-PD-1 Ab treatment (PD-1 group), 31 (34.4%) patients received anti-PD-1 Ab in combination with TKIs therapy (PD-1 + TKI group), 8 (8.9%) patients received anti-PD-1 Ab treatment in combination with TACE (PD-1 + TACE group), and 15 (16.7%) patients received anti-PD-1 Ab treatment, TKIs therapy and TACE (PD-1 + TKI + TACE group). Additionally, 82 (91.1%) patients were adult males, 44 (48.9%) patients were older than 55 years, 77 (85.6%) patients were infected with hepatitis B virus (HBV).

Subsequently, the pathological morphology of surgical specimens from the 90 patients was examined based on the HE slides. 46 (51.1%) cases displayed a complete tumor capsule, 50 (55.6%) cases had liver capsule invasion, and MVI was observed in 52 (57.8%) cases. Additional details are provided in Table 1.

**Table 1.** Clinicopathological features of 90 HCC patients.

Clinicopathology	All patients N = 90(%)	Clinicopathology	All patients N = 90(%)
**Sex**		Liver cirrhosis	
** Male**	82(91.1)	Yes	41(45.6)
** Female**	8(8.9)	No	49(54.4)
**Age**		Incisal margin	
** ≤55 years**	46(51.1)	Negative	90(100.0)
** >55 years**	44(48.9)	Positive	0(0.0)
**HBV^a^**		Necrosis	
** Yes**	77(85.6)	Yes	72(80)
** No**	13(14.4)	No	18(20)
**Antiviral therapy**		TILs^d^	
** Yes**	59(65.5)	Yes	46(51.1)
** No**	31(34.4)	No	44(48.9)
**Single focal**		TLSs^e^	
** Yes**	58(64.4)	Yes	16(17.8)
** No**	32(35.6)	No	74(82.2)
**CNLC^b^ staging**		Fibrillation	
** Stage Ia**	28(31.1)	Yes	73(81.1)
** Stage Ib**	31(34.4)	No	17(18.9)
** Stage IIa**	13(14.4)	Blood	
** Stage IIb**	7(7.8)	Yes	20(22.2)
** Stage IIIa**	11(12.2)	No	70(77.8)
**Differentiation**		Foam cell	
** Level II**	42(46.7)	Yes	35(38.9)
** Level III**	37(41.1)	No	55(61.1)
** /^j^**	11(12.2)	Cholesterol crystal	
**Liver capsule invasion**		Yes	12(13.3)
** Yes**	50(55.6)	No	78(86.7)
** No**	40(44.4)	Surgery	
**MVI^c^**		Partial hepatectomy	78(86.7)
** M0**	52(57.8)	Liver transplantation	12(13.3)
** M1**	23(25.6)	Neoadjuvant Therapy	
** M2**	15(16.7)	P^f^	16(17.8)
**Tumor capsule**		P+TA^g^	8(8.9)
** Complete**	46(51.1)	TA^h^	20(22.2)
** Incomplete**	44(48.9)	P+TK+TA^i^	15(16.7)
		P+TK^j^	31(34.4)

a Hepatitis B virus.

b Cina Liver Cancer Staging.

c Microvascular invasion.

d Tumor infiltrating lymphocytes.

e Tertiary lymphoid structures.

f PD-1 group.

g PD-1+TACE group.

h TACE group.

i PD-1+TKI+TACE group.

j PD-1+TKI group.

j Tumors have completely regressed.

### Treatment-related histopathological features

**Figure 1.**
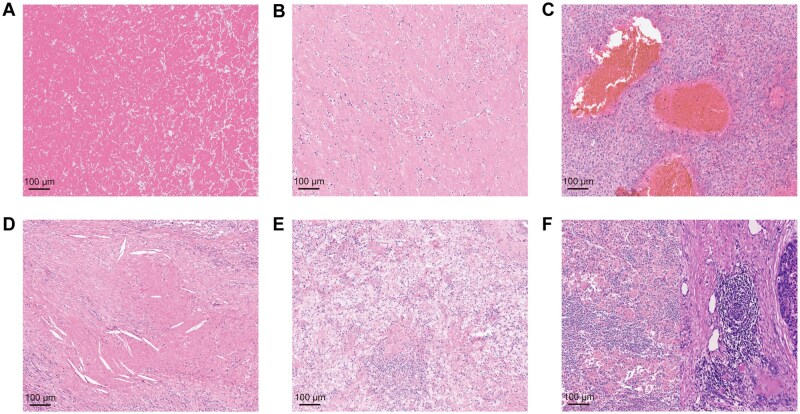
**Histopathological features and pathological remission of HCC patients treated with neoadjuvant therapy**. (A) Necrosis. (B) Fibrosis. (C) Bleeding. (D) Cholesterol crystallization. (E) Foam cell response. (F) Tumor-infiltrating lymphocytes (TILs) and tertiary lymphoid structures (TLSs).

Moreover, we observed various treatment-related histopathological characteristics, including necrosis, fibrosis, bleeding, foam cell response, appearance of cholesterol crystallization, TILs, and TLSs (Figure 1A–F). Since the majority of patients receiving immunotherapy were also undergoing TKI therapy or TACE, we initially analyzed histopathological features between different treatment groups. The results revealed no statistically significant differences in various histological characteristics between the PD-1 group and the PD-1 + TACE group or between the PD-1 group and the PD-1 + TKI group. However, statistically significant differences were observed in TILs, foam cell response, and cholesterol crystallization between the PD-1 + TACE group and the TACE group (p = 0.003, p = 0.004, p = 0.026). Furthermore, a statistically significant difference in TILs and foam cell response was observed between the PD-1 + TKI + TACE group and the TACE group (p = 0.014, p = 0.036). These findings suggest that HCC patients treated with anti-PD-1 Ab treatment prior to surgery may exhibit increased TILs and foam cell response (Supplemental Table 1).

In addition to the histopathological observations mentioned earlier, we also assessed the viable tumor on all slides of each case. Previous studies have established multidisciplinary recommendations for pathological assessment of solid tumor resection specimens following neoadjuvant therapy, such as lung cancer and melanoma, which defined a viable tumor cell percentage of ≤ 10% as mPR. Additionally, these studies found that the overall postoperative survival of patients with mPR is significantly better than that of non-mPR patients.^15^^,^^16^ However, a clear and universally accepted standard for mPR cutoff values in HCC resection specimens following neoadjuvant therapy has not yet to be established.

**Figure 2.**
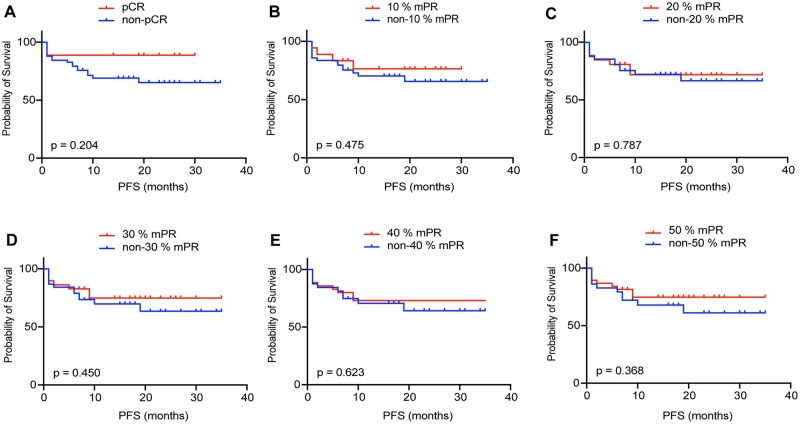
**Relationship between different mPR thresholds and postoperative** R**FS in HCC patients with neoadjuvant immunotherapy**. **(**A-F) Kaplan–Meier curves for RFS based on the pCR and the different mPR thresholds.

Therefore, in our study of 70 HCC patients receiving neoadjuvant immunotherapy, we attempted to define pCR (Supplemental Figure 1A) and different mPR cutoff values (10%, 20%, 30%, 40%, and 50%; Supplemental Figure 1B–C). We conducted further analysis based on the RFS and OS of the patients, and our findings revealed that there was no statistically significant difference in postoperative RFS and OS among patients with different mPR cutoff values (Figure 2A–F). We also conduct a statistical analysis of the treatment-related clinicopathological features within different mPR cutoff values. It was astonishing to discover that foam cell response, TILs and TLSs were significantly higher in the cases achieving mPR than in non-mPR cases, regardless of the mPR cutoff values (p < 0.05).

### Construction of a nomogram model to predict early recurrence in HCC patients following neoadjuvant immunotherapy

To further investigate the impact of foam cell response, TILs, TLSs, in the tumor microenvironment on the early recurrence of HCC patients following neoadjuvant therapy, we conducted an analysis using IHC staining related to the classification of immune cells on 70 cases. Then, we counted the positive immune cell numbers in CT, IM, and NL regions (Supplemental Figure 2A–B), The detailed data on the density of each immune marker are available in Supplemental Material 2, and categorized cases into high and low immune cell infiltration groups based on the median count of positive immune cells, Representative images of high and low infiltration densities for each immune marker are provided in Supplemental Material 3.

**Figure 3.**
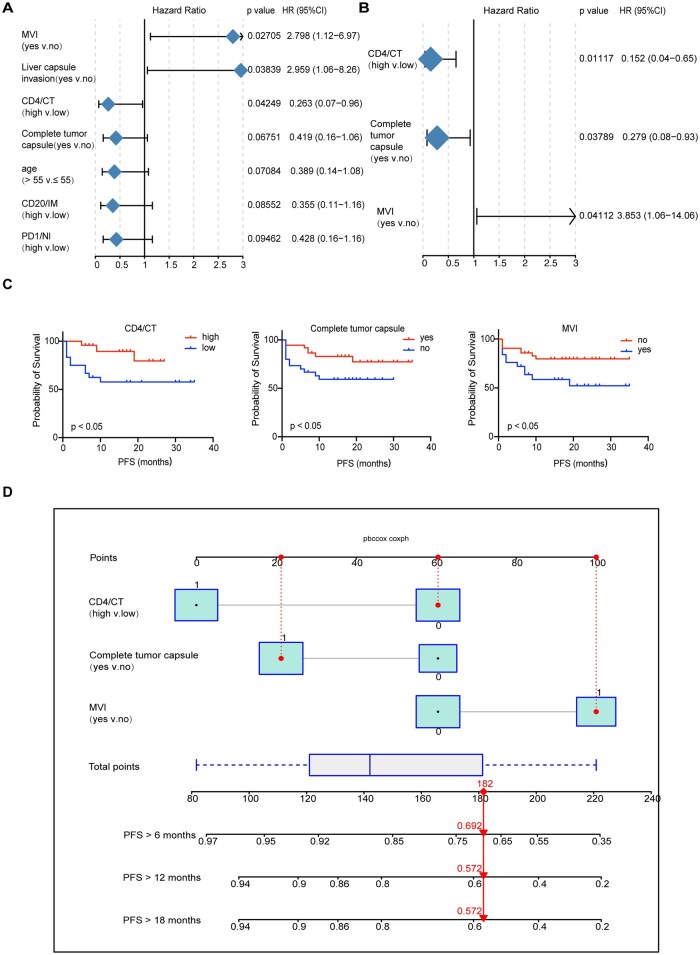
**Nomogram model to predict early recurrence in HCC patients after neoadjuvant immunotherapy**. (A) Cox univariate analysis of clinicopathological and immunological factors. (B) Cox multivariate analysis of the factors identified with a P value < 0.1 in the Cox univariate analysis. (C) Kaplan–Meier curves for RFS based on the independent risk factors. (D) Nomogram model based on the independent risk factors.

By conducting Cox univariate analysis on clinical-pathology-immune factors (Supplemental Table 2), we identified the presence of MVI, incomplete tumor capsule, and low CD4^+^ T cell infiltration in the CT region as risk factors which decreased RFS (Figure 3A). Subsequently, we performed Cox multivariate analysis on the factors identified with a *P* value < 0.1 in the Cox univariate analysis. This analysis revealed that the presence of MVI, incomplete tumor capsule, and low CD4^+^ T cell infiltration in the CT region also were independent risk factors for decreased RFS (Figure 3B–C).

Finally, to improve the prediction of postoperative recurrence in HCC patients following neoadjuvant immunotherapy, we integrated MVI, tumor capsule, and CD4^+^ T cell density of the CT region to construct a nomogram model (Figure 3D). The model exhibited a C-index of 0.826 (0.712-0.940), indicating good discriminatory ability.

### Evaluation of the nomogram prediction model

**Figure 4.**
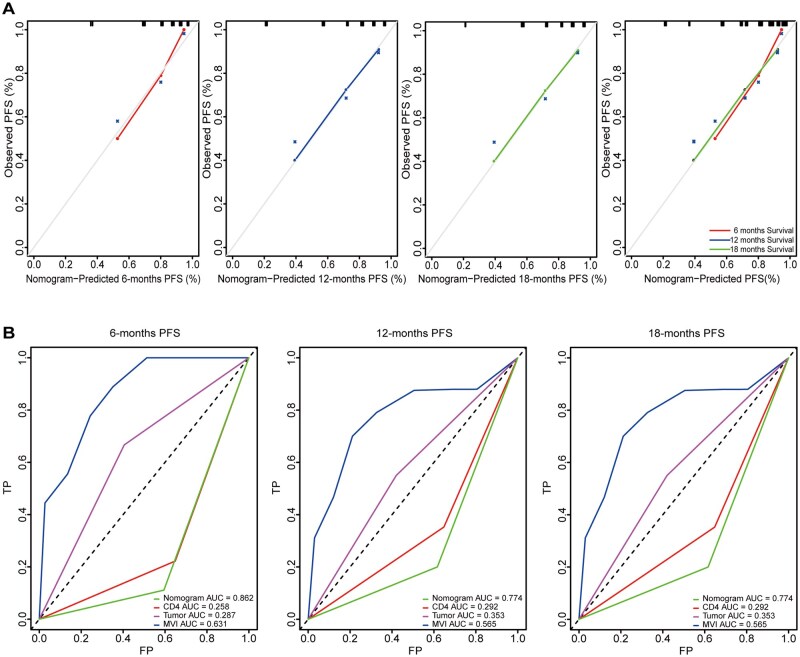
**Evaluation of the nomogram prediction model**. (A) Calibration curves of patients’ RFS at 6, 12, and 18 months after surgery. (B) ROC curve analysis for different groups.

We conducted further analysis to assess the predictive performance of the model. We examined the calibration curves of patients’ RFS at 6, 12, and 18 months following surgery. The results demonstrated a high level of agreement between the predicted values from the model and the actual outcomes (Figure 4A). To further evaluate the predictive efficacy, we utilized ROC curve analysis, and compared the performance of the nomogram model with three independent indicators (MVI, tumor capsule, and CD4^+^ T cell density of the CT region) at 6, 12, and 18 months postoperative. The area under the curve (AUC) of the nomogram model was significantly higher to that of these three independent indicators (Figure 4B). These findings strongly support the notion that the nomogram model, based on these three independent indicators, exhibits superior predictive capabilities.

Furthermore, we conducted an experiment in which we removed each of these three independent risk factors from the nomogram model, namely, model 1 without the MVI model 2 without the complete tumor capsule, and model 3 without CD4^+^ T cell density of the CT region. Subsequently, we compared the nomogram model with model 1, 2, and 3 utilizing the NRI and IDI values. Interestingly, we observed that the IDI values were consistently greater than 0, with the largest difference observed when comparing the nomogram model to model 3. These results clearly indicate that the nomogram model possesses better predictive power than models that exclude any one of three independent risk factors. Moreover, the CD4^+^ T cell density of the CT region significantly enhanced the predictive capabilities of the model (Supplemental Table 3).

**Figure 5.**
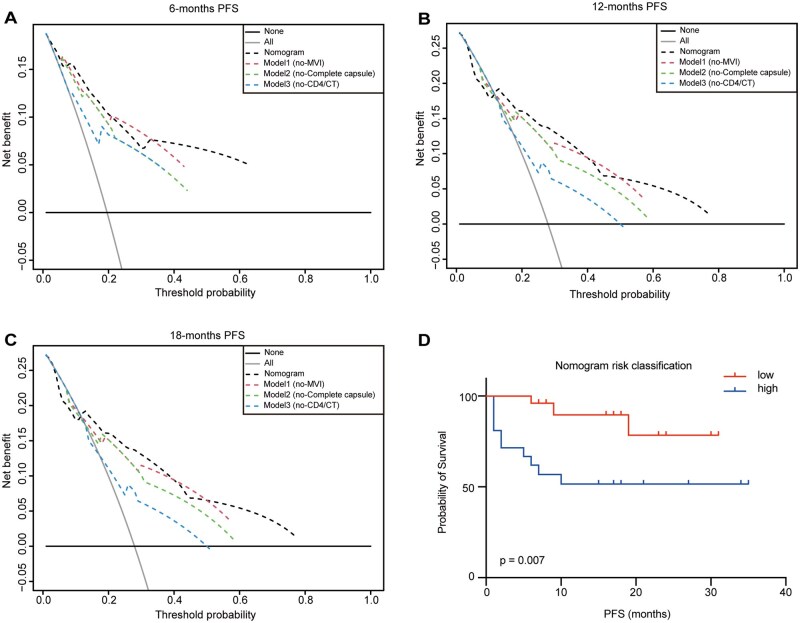
**Evaluation of the nomogram prediction model**. (A-C) DCA assesses the impact of three independent risk factors on the model. (D) Kaplan-Meier curves for RFS were generated according to the nomogram model.

In addition, we conducted DCA to evaluate the impact of three independent risk factors in the nomogram model. The results revealed that the DCA curve of the nomogram model deviated significantly from the reference curve, whereas the curve of model 3 closely resembled the reference curve. This finding suggests that the nomogram model exhibits the highest net benefit and superior predictive capability. However, excluding the CD4^+^ T cell density in the CT region from model 3 led to a noticeable reduction in its predictive power. This underscores the critical role of CD4^+^ T cell density of the CT region for accurate prediction of RFS (Figure 5A-C).

Subsequently, we categorized 70 HCC patients into high-risk group and low-risk group based on the risk classification generated by the nomogram model. Kaplan–Meier survival analysis demonstrated that the low-risk group exhibited significantly longer RFS than the high-risk group (p = 0.007; Figure 5D).

## Discussion

HCC remains one of the most lethal malignancies, with high mortality and recurrence rates. Despite surgical resection being the preferred curative treatment for HCC, early recurrence rates remain unacceptably high, with up to 70% of patients experiencing relapse within the first two years postoperatively.^17^^,^^18^ In recent years, combination therapies, such as ICIs with TKIs, have shown promising outcomes in patients with advanced HCC.^6^^,^^19^ However, even with neoadjuvant immunotherapy, which aims to improve surgical outcomes, the recurrence rates are still suboptimal, necessitating more accurate predictive models to guide treatment decisions and improve patient outcomes.

In several cancer types, including lung cancer and melanoma, mPR has been proposed as a reliable endpoint to assess the efficacy of neoadjuvant therapies.^20^^,^^21^ For example, in lung cancer, a 10% mPR threshold has been established based on robust clinical trial data and expert consensus, particularly in the context of neoadjuvant chemotherapy.^22^ However, in the context of HCC, there is no clear consensus on the mPR threshold, with varying cutoff values ranging from 10% to 50% across different studies.^23–25^ This variability highlights the need for standardization, as the determination of an appropriate mPR cutoff is crucial for predicting patient prognosis after neoadjuvant immunotherapy. In our study, we tested several mPR cutoff values (10%, 20%, 30%, 40%, 50%) and also evaluated the pCR to determine their prognostic significance in relation to RFS. Unfortunately, none of these cutoff values achieved statistical significance, indicating that further research is needed to determine the optimal mPR threshold in HCC patients receiving neoadjuvant immunotherapy. Recently, a study has demonstrated that the degree of tumor regression following neoadjuvant ICI therapy serves as a reliable surrogate endpoint for RFS in patients with HCC post-surgery. The optimal threshold for pathological response is believed to be 90%.^26^

Beyond mPR analysis, we also constructed a nomogram model incorporating MVI, tumor capsule integrity, and CD4^+^ T cell density in the CT region. This is the first model to predict early postoperative recurrence in HCC following neoadjuvant immunotherapy by integrating regional immune markers with clinicopathological features. Similar to our findings, previous studies have also identified MVI[10, 11] and the incomplete tumor capsule^27^ as crucial factors influencing early recurrence in HCC patients post-surgery. Moreover, recent research has suggested a pivotal role of CD4^+^ T cells in determining the efficacy of immunotherapy in HCC.^28^^,^^29^ For instance, studies have shown that even a small number of CD4^+^ T cells can eradicate tumors that evade direct CD8^+^ T cell targeting, with CD4^+^ effector T cells clustering predominantly at tumor-invasive margins.^29^ However, our study uniquely highlights that higher CD4^+^ T cell density in the CT region is associated with longer RFS in patients treated with neoadjuvant immunotherapy. The precise mechanism underlying this correlation remains unclear and warrants further investigation. Overall, our findings provide valuable real-world data on the tumor immune microenvironment in HCC patients receiving neoadjuvant immunotherapy. The importance of MVI, tumor capsule integrity, and CD4^+^ T cell density in predicting early postoperative recurrence is emphasized. These factors can serve as critical markers for stratifying patient risk and guiding postoperative surveillance strategies.

In conclusion, while neoadjuvant immunotherapy shows promise for HCC, more research is needed to refine predictive models. The nomogram model developed in our study effectively predicts early recurrence after surgery and can help clinicians take timely measures to improve outcomes. Expanding sample sizes and continuously optimizing such predictive models will be crucial to enhancing decision-making and prolonging RFS in HCC patients undergoing neoadjuvant immunotherapy.

## Authors’ contributions

Yanrui Pang (Conceptualization, Data curation, Formal analysis, Funding acquisition, Methodology, Software, Writing—original draft, Writing—review & editing), Xuelian Zhao(Formal analysis, Methodology, Resources), Xinxin Guo(Data curation, Methodology, Resources), and Jing Han(Conceptualization, Data curation, Formal analysis, Investigation, Methodology, Supervision, Writing—original draft, Writing—review & editing), Yuan Ji(Conceptualization, Data curation, Funding acquisition, Investigation, Project administration, Resources, Supervision, Visualization, Writing—original draft, Writing—review & editing).

## Supplementary Material

oyaf368_Supplementary_Data

## Data Availability

The datasets generated during and/or analysed during the current study are available from the corresponding author on reasonable request.
