# Supplementary material for: An early relapse prediction model based on pathological features following neoadjuvant immunotherapy for hepatocellular carcinoma
Source: Oncologist. 2025 Nov 10;31(1):oyaf368. doi: 10.1093/oncolo/oyaf368 (PMC12771520; doi:10.1093/oncolo/oyaf368)
Supplement: oyaf368_Supplementary_Data [file oyaf368_supplementary_data.zip › Supplemental Table 2.docx]

| **Supplemental Table 2. Cox Univariate Analysis of All Included Factors.** | | | | |
| --- | --- | --- | --- | --- |
| Factor | HR | HR.95L | HR.95H | pvalue |
| MVI (yes v.no) | 2.79849866 | 1.12384367 | 6.968580206 | 0.027050442 |
| Capsule.invasion (yes v.no) | 2.959072688 | 1.059707042 | 8.262765866 | 0.038391374 |
| CD4 (CT) (high v.low) | 0.262596483 | 0.072154124 | 0.955689138 | 0.042485978 |
| Tumor.capsular (yes v.no) | 0.418596941 | 0.164570839 | 1.064729332 | 0.067506621 |
| Age (>55.1 v.<55.1) | 0.389419714 | 0.13997475 | 1.083393352 | 0.070835046 |
| CD20 (IM) (high v.low) | 0.355205954 | 0.109169085 | 1.155741752 | 0.085521889 |
| PD1 (NL) (high v.low) | 0.427679745 | 0.157973676 | 1.157850909 | 0.09461676 |
| PD1 (CT) (high v.low) | 0.429849005 | 0.143898498 | 1.284031249 | 0.130483039 |
| PD1 (IM) (high v.low) | 0.441059904 | 0.1474297 | 1.319502371 | 0.143173338 |
| CNLC | 1.954841229 | 0.793972145 | 4.813020524 | 0.144809861 |
| CD4 (IM) (high v.low) | 0.488549443 | 0.159262038 | 1.49866573 | 0.210375921 |
| PDL1(22C3) (high v.low) | 0.442592173 | 0.121639331 | 1.610398783 | 0.216120245 |
| CD68 (CT) (high v.low) | 2.02129295 | 0.659379792 | 6.196163788 | 0.218208576 |
| ALT (>40 v.<40) | 0.547130117 | 0.207838113 | 1.440310248 | 0.222025894 |
| CD206 (CT) (high v.low) | 1.833984982 | 0.598907299 | 5.616062656 | 0.288164872 |
| CD20 (CT) (high v.low) | 0.548785589 | 0.179305121 | 1.679626444 | 0.293093168 |
| CD38 (NL) (high v.low) | 0.604745052 | 0.230078826 | 1.58952731 | 0.307704962 |
| AST (>35 v.<35) | 0.996806379 | 0.990680938 | 1.002969694 | 0.309108308 |
| HBsAg (yes v.no) | 2.832327252 | 0.377982166 | 21.22342899 | 0.310982632 |
| gender.male (yes v.no) | 0.53444817 | 0.155589796 | 1.835819919 | 0.319690551 |
| CD38 (CT) (high v.low) | 0.570327722 | 0.18641403 | 1.7448993 | 0.325002457 |
| CD4 (NL) (high v.low) | 0.6128987 | 0.226257109 | 1.660256417 | 0.335620439 |
| FOXP3 (IM) (high v.low) | 0.591416543 | 0.193153255 | 1.810860129 | 0.357606671 |
| CD38 (IM) (high v.low) | 1.688261591 | 0.550709301 | 5.175556674 | 0.359533418 |
| CD15 (CT) (high v.low) | 1.675469549 | 0.54588724 | 5.142450685 | 0.367062059 |
| PDL1.288 (>0.1 v.<0.1) | 0.878890815 | 0.663388668 | 1.164398944 | 0.368401996 |
| CD3 (IM) (high v.low) | 0.598225612 | 0.193794809 | 1.84666392 | 0.371646465 |
| CD15 (NL) (high v.low) | 1.467052919 | 0.557873748 | 3.857941471 | 0.437218097 |
| PDL1.22C3 (>1 v.<1) | 0.941835016 | 0.807726618 | 1.098209689 | 0.44449558 |
| The.number.of.tumors (>1 v.1) | 1.401884219 | 0.569223824 | 3.452559922 | 0.462572674 |
| CD8 (CT) (high v.low) | 0.675026049 | 0.21354122 | 2.13382769 | 0.503325192 |
| CD8 (IM) (high v.low) | 0.680130189 | 0.213340796 | 2.168254183 | 0.514632172 |
| AFP (>200 v.<200) | 0.9998953 | 0.999545575 | 1.000245147 | 0.557446749 |
| CD68 (IM) (high v.low) | 1.384191303 | 0.46123467 | 4.154036304 | 0.562027259 |
| antiviral.therapy (yes v.no) | 1.322314361 | 0.474809223 | 3.682563824 | 0.592905854 |
| CD20 (NL) (high v.low) | 0.772747715 | 0.29754461 | 2.006889089 | 0.596505712 |
| CD3 (NL) (high v.low) | 1.291903706 | 0.495655008 | 3.367292087 | 0.600284327 |
| FOXP3 (CT) (high v.low) | 1.310493382 | 0.437965494 | 3.921297293 | 0.628704239 |
| CD15 (IM) (high v.low) | 0.786661967 | 0.262190952 | 2.360253263 | 0.668616773 |
| CD68 (NL) (high v.low) | 1.224572001 | 0.471227879 | 3.182274763 | 0.677571063 |
| CD8 (NL) (high v.low) | 0.812330793 | 0.301745607 | 2.186879621 | 0.680812665 |
| FOXP3 (NL) (high v.low) | 1.20414247 | 0.443727763 | 3.267677188 | 0.715325145 |
| CD206 (IM) (high v.low) | 0.8320978 | 0.278327316 | 2.487670846 | 0.742192475 |
| Cirrhosis (yes v.no) | 0.862653185 | 0.346812873 | 2.145740759 | 0.750651637 |
| CD206 (NL) (high v.low) | 1.127416577 | 0.434747629 | 2.923691936 | 0.805163827 |
| CD3 (CT) (high v.low) | 1.126915529 | 0.376852137 | 3.369859111 | 0.830709277 |
| Hepatitis.B (yes v.no) | 0.949020882 | 0.276404835 | 3.258411285 | 0.933743082 |
