# Supplementary material for: An early relapse prediction model based on pathological features following neoadjuvant immunotherapy for hepatocellular carcinoma
Source: Oncologist. 2025 Nov 10;31(1):oyaf368. doi: 10.1093/oncolo/oyaf368 (PMC12771520; doi:10.1093/oncolo/oyaf368)
Supplement: oyaf368_Supplementary_Data [file oyaf368_supplementary_data.zip › Supplemental Table 3.docx]

| **Supplemental Table 3. IDI and NRI evaluation of the predictive performance of nomogram models** | | | | |
| --- | --- | --- | --- | --- |
| Nomogram vs. Model1/2/3 | IDI^a^ | P value | NRI^b^ | P value |
| 6-months PFS |  | | | |
| Model1 | 0.057 | 0.135 | 0.340 | **0.023** |
| Model2 | 0.065 | 0.069 | 0.417 | **0.005** |
| Model3 | 0.129 | **0.004** | 0.525 | **0.009** |
| 12-months PFS |  | | | |
| Model1 | 0.064 | 0.140 | 0.340 | **0.022** |
| Model2 | 0.074 | 0.074 | 0.382 | **0.031** |
| Model3 | 0.154 | **0.004** | 0.525 | **0.009** |
| 18-months PFS |  | | | |
| Model1 | 0.064 | 0.140 | 0.340 | **0.023** |
| Model2 | 0.074 | 0.074 | 0.382 | **0.031** |
| Model3 | 0.154 | **0.004** | 0.525 | **0.009** |
| ^a^ integrated discrimination improvement. ^b^ net reclassification improvement. | | | | |
