# Supplementary material for: An early relapse prediction model based on pathological features following neoadjuvant immunotherapy for hepatocellular carcinoma
Source: Oncologist. 2025 Nov 10;31(1):oyaf368. doi: 10.1093/oncolo/oyaf368 (PMC12771520; doi:10.1093/oncolo/oyaf368)
Supplement: oyaf368_Supplementary_Data [file oyaf368_supplementary_data.zip › Supplemental Material 3.pdf]

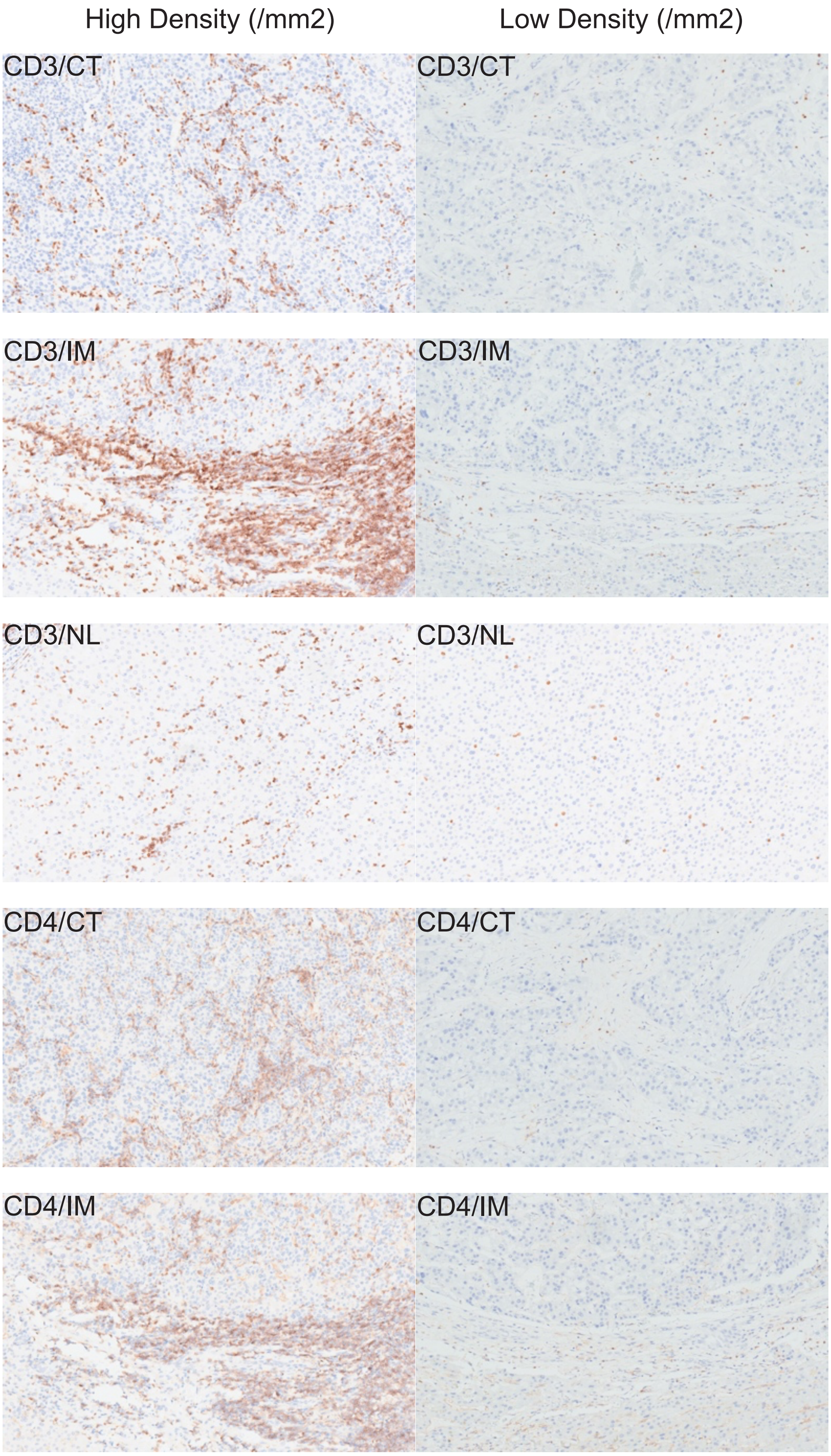
High Density (/mm2)
Low Density (/mm2)
CD3/CT
CD3/CT
CD3/IM
CD3/IM
CD3/NL
CD3/NL
CD4/CT
CD4/CT
CD4/IM
CD4/IM

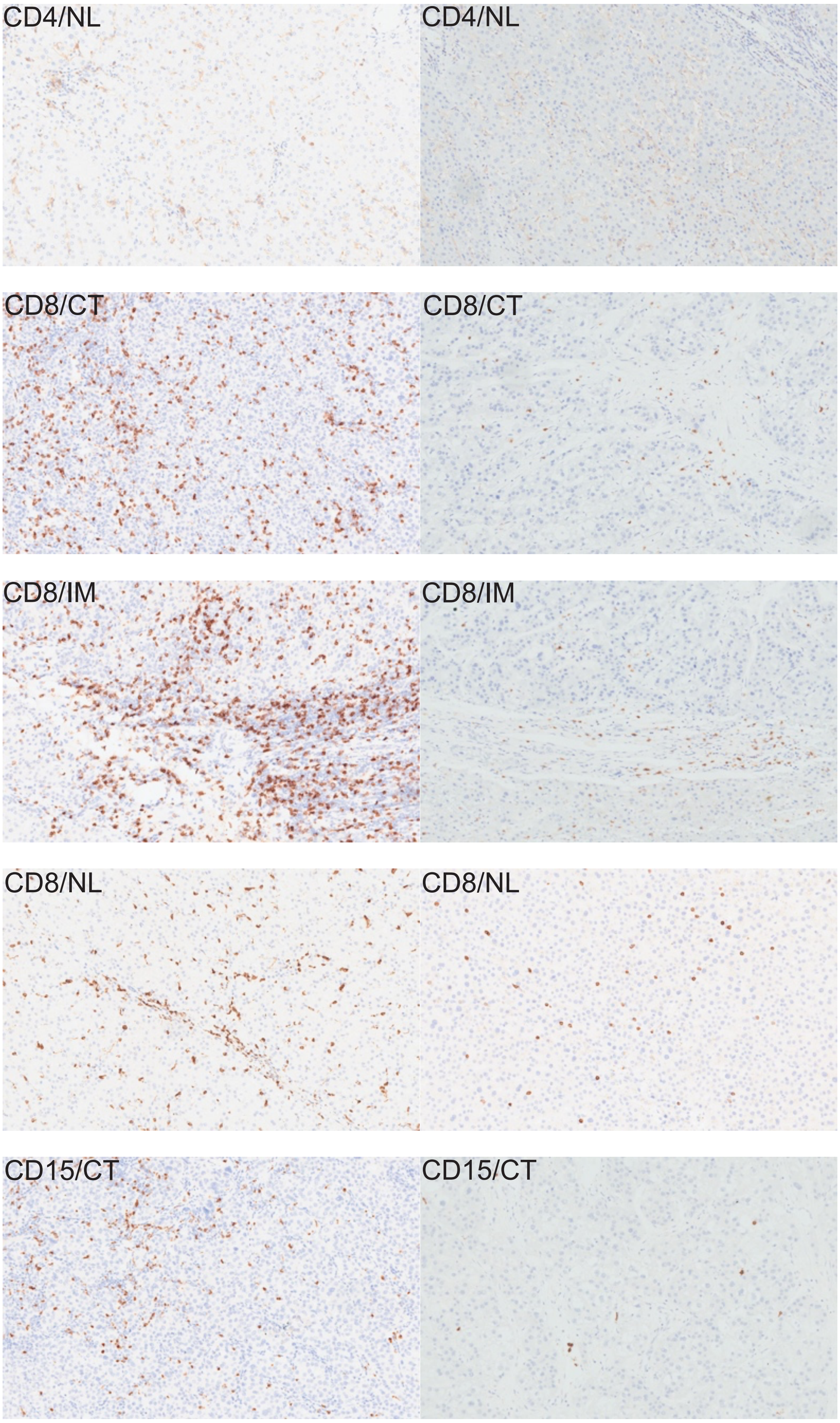
CD4/NL
CD4/NL
CD8/CT
CD8/CT
CD8/IM
CD8/IM
CD8/NL
CD8/NL
CD15/CT
CD15/CT

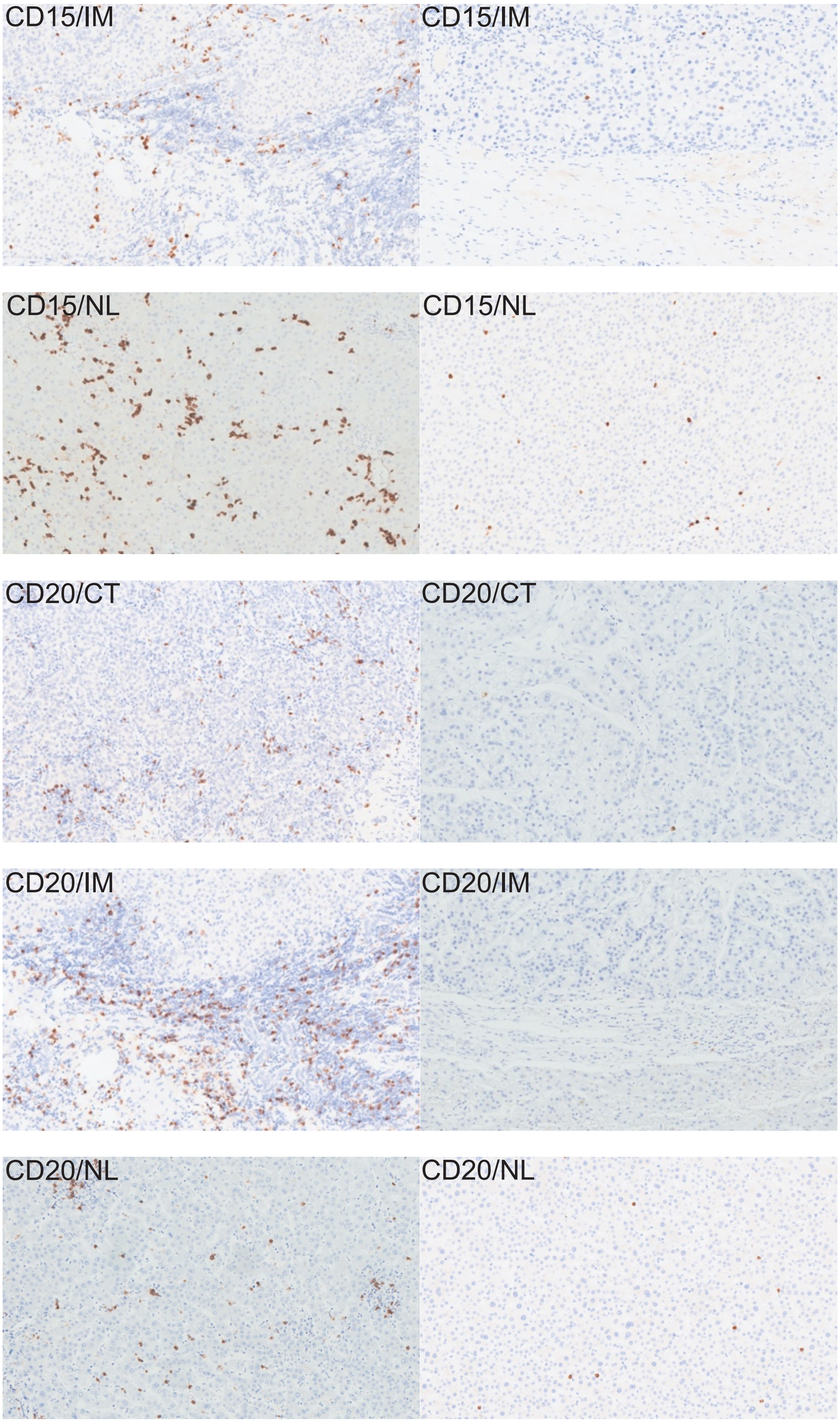
CD15/IM
CD15/IM
CD15/NL
CD15/NL
CD20/CT
CD20/CT
CD20/IM
CD20/IM
CD20/NL
CD20/NL

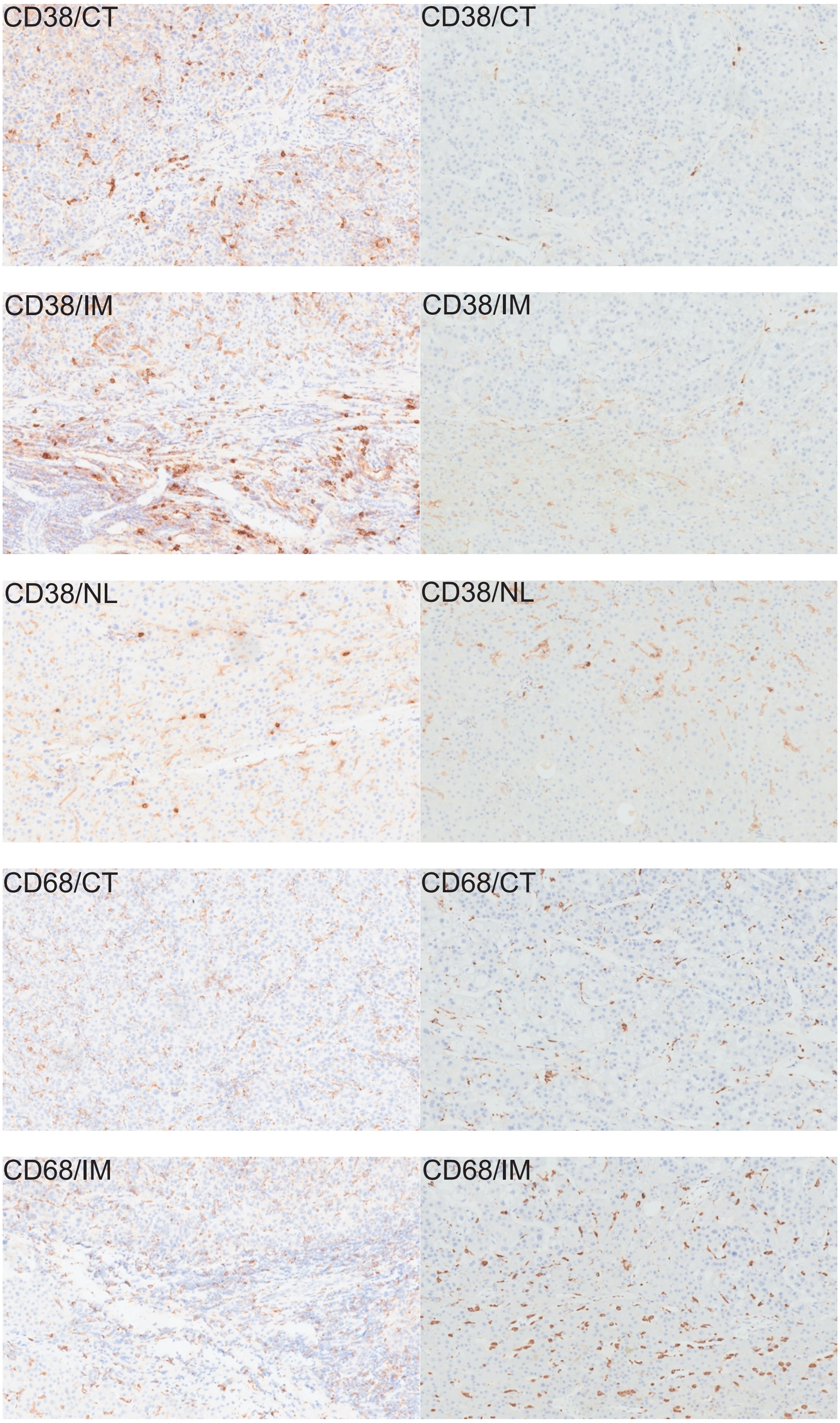
CD38/CT
CD38/CT
CD38/IM
CD38/IM
CD38/NL
CD38/NL
CD68/CT
CD68/CT
CD68/IM
CD68/IM

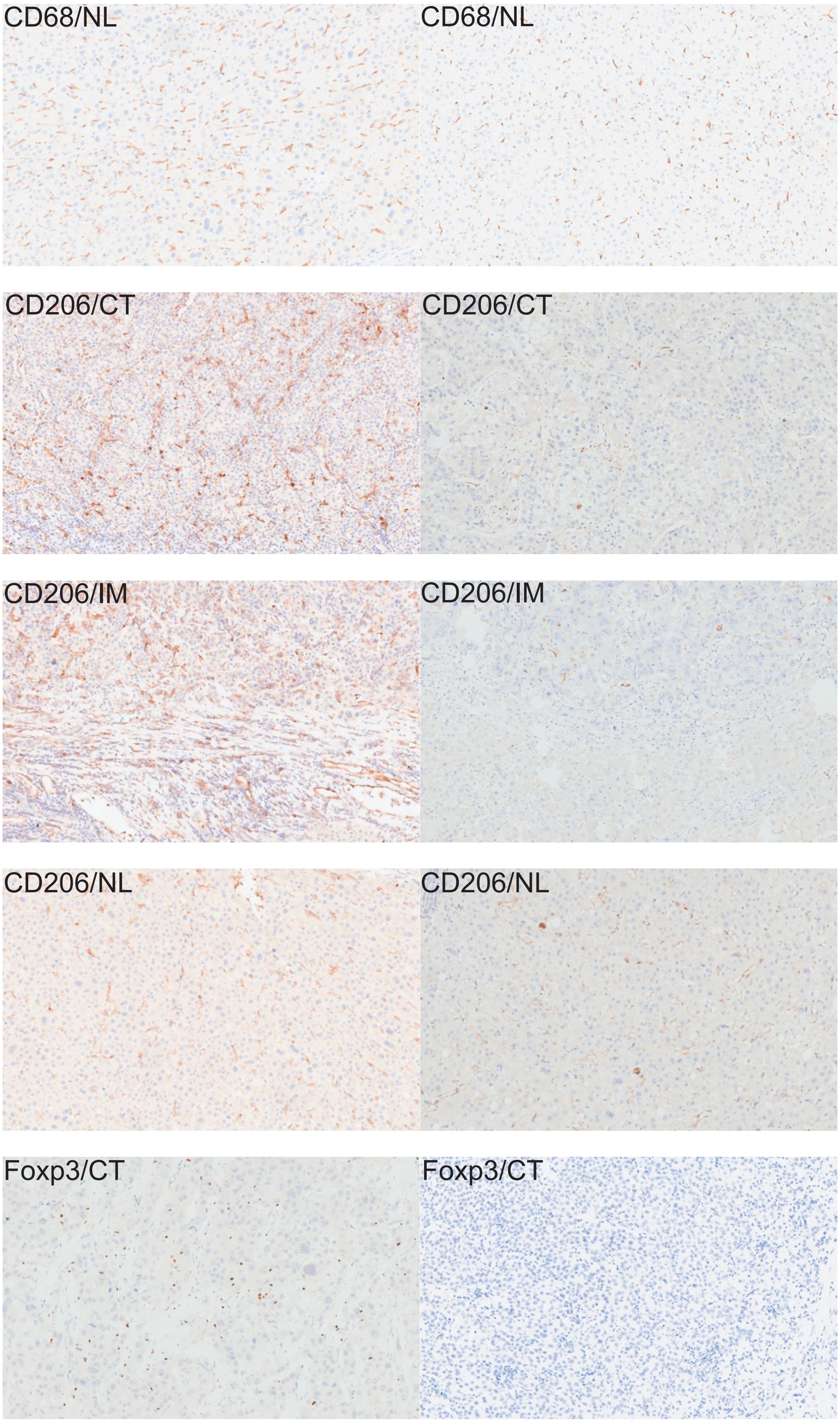
CD68/NL
CD68/NL
CD206/CT
CD206/CT
CD206/IM
CD206/IM
CD206/NL
CD206/NL
Foxp3/CT
Foxp3/CT

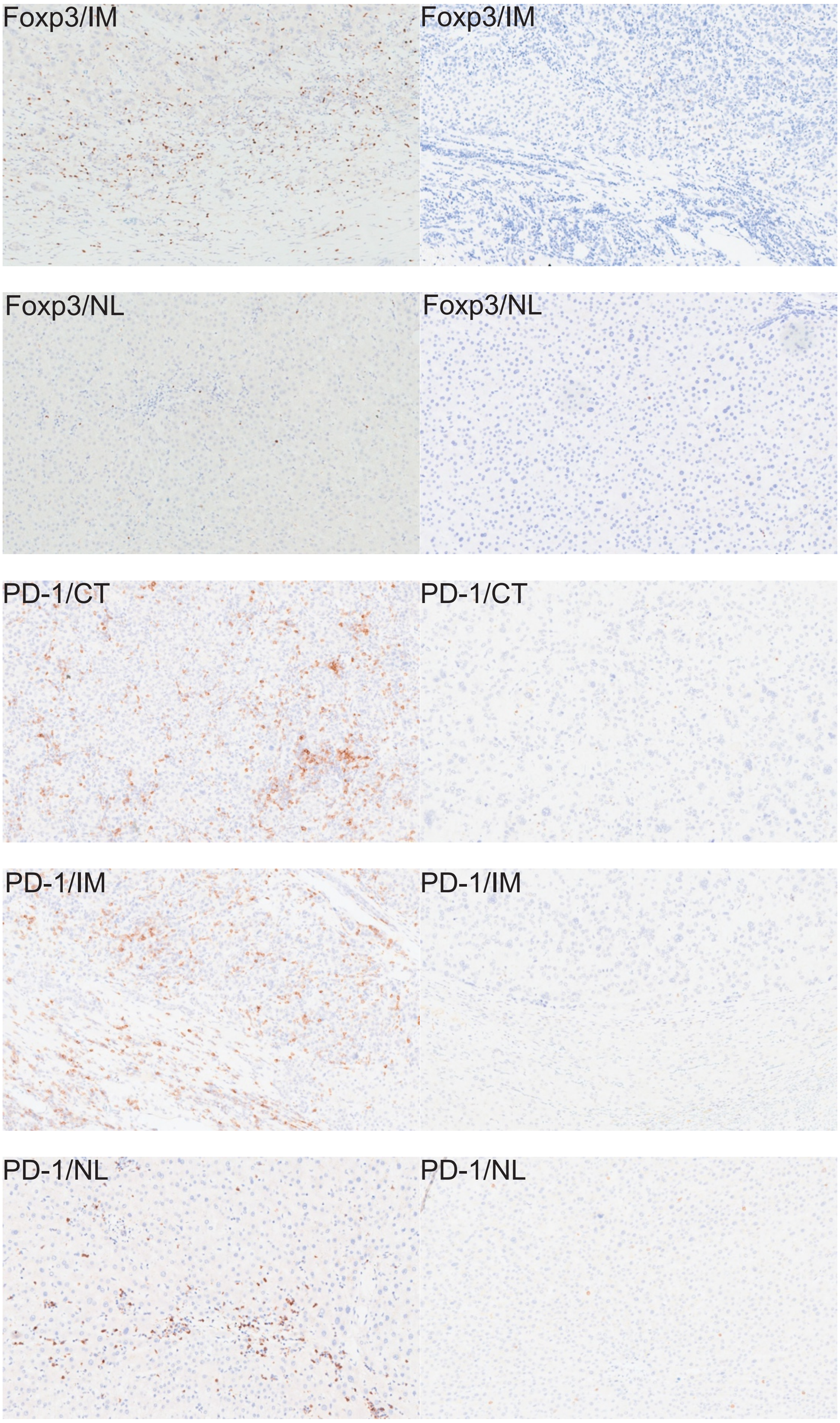
Foxp3/IM
Foxp3/IM
Foxp3/NL
Foxp3/NL
PD-1/CT
PD-1/CT
PD-1/IM
PD-1/IM
PD-1/NL
PD-1/NL
