# Supplementary material for: An early relapse prediction model based on pathological features following neoadjuvant immunotherapy for hepatocellular carcinoma
Source: Oncologist. 2025 Nov 10;31(1):oyaf368. doi: 10.1093/oncolo/oyaf368 (PMC12771520; doi:10.1093/oncolo/oyaf368)
Supplement: oyaf368_Supplementary_Data [file oyaf368_supplementary_data.zip › Supplemental Figure Legends.docx]

**Supplemental Figure 1. Pathological remission of HCC patients treated with neoadjuvant therapy (low-power field)**

(A) Pathological complete response (pCR). No residual tumor was found, and the regression bed component was fibrosis with minor bleeding.

(B) 30% mPR, the regression bed component was necrosis.

(C) 50% mPR, the regression bed component was necrosis and bleeding.

The red thread wraps around the residual tumor, and the black thread wraps around the regression bed.

**Supplemental Figure 2. Immune microenvironment in HCC patients with neoadjuvant immunotherapy**

(A) Schematic diagram of HCC pathological section division. An area of 0.5 mm inward and outward from the tumor boundary was used as the invasive margin (IM). The tumor area within the IM is the central tumor (CT), and the peripheral liver area outside the IM is considered the normal liver (NL).

(B) Regionalization of CD4 immunohistochemical staining images.

**Supplemental Figure 3. Immune Cell Expression Differences: Comparative Analysis in CT, IM, and NL Regions.**

**Supplemental Material 3. Representative Images of High and Low Immune Cell Infiltration Across Different Regions.**
