# Supplementary material for: An early relapse prediction model based on pathological features following neoadjuvant immunotherapy for hepatocellular carcinoma
Source: Oncologist. 2025 Nov 10;31(1):oyaf368. doi: 10.1093/oncolo/oyaf368 (PMC12771520; doi:10.1093/oncolo/oyaf368)
Supplement: oyaf368_Supplementary_Data [file oyaf368_supplementary_data.zip › Supplemental Material 1.docx]

| **Supplemental Material 1: Medication information of the 70 patients.** | | | | | | | | | |
| --- | --- | --- | --- | --- | --- | --- | --- | --- | --- |
| **No.** | **Gender** | **Age** | **Treatment Modality** | **PD-1 Relative Time (days)** | **TACE Relative Time (days)** | **TKI Relative Time (days)** | **anti-PD1 agent** | **anti-PD1 dose** | **cycles of anti-PD1** |
| 1 | Female | 66 | PD-1 | -21 | / | / | Toripalimab | 480mg | 1 |
| 2 | Male | 50 | PD-1 | -185 | / | / | Pembrolizumab | 200mg | q3w; 8 |
| 3 | Male | 40 | PD-1 | -54 | / | / | Camrelizumab | 200mg | q3w; 2 |
| 4 | Male | 51 | PD-1 | -59 | / | / | Toripalimab | 480mg | 1 |
| 5 | Male | 67 | PD-1 | -47 | / | / | Toripalimab | 480mg | 1 |
| 6 | Male | 45 | PD-1 | -120 | / | / | Camrelizumab | 200mg | q3w; 3 |
| 7 | Female | 58 | PD-1 | -23 | / | / | Toripalimab | 480mg | 1 |
| 8 | Male | 52 | PD-1 | -23 | / | / | Toripalimab | 480mg | 1 |
| 9 | Male | 43 | PD-1 | -24 | / | / | Toripalimab | 480mg | 1 |
| 10 | Male | 55 | PD-1 | -167 | / | / | Camrelizumab | 200mg | q3w; 6 |
| 11 | Female | 50 | PD-1 | -95 | / | / | Camrelizumab | 200mg | q3w; 3 |
| 12 | Male | 52 | PD-1 | -24 | / | / | Toripalimab | 480mg | 1 |
| 13 | Male | 50 | PD-1 | -291 | / | / | Camrelizumab | 200mg | q3w; 8 |
| 14 | Male | 56 | PD-1 | -182 | / | / | Camrelizumab | 200mg | q3w; 6 |
| 15 | Male | 54 | PD-1 | -25 | / | / | Toripalimab | 480mg | 1 |
| 16 | Male | 56 | PD-1 | -29 | / | / | Toripalimab | 480mg | 1 |
| 17 | Male | 69 | PD-1+TACE | -164 | -356 | / | Camrelizumab | 200mg | q3w; 6 |
| 18 | Male | 49 | PD-1+TACE | -200 | -234 | / | Nivolumab | 200mg | q3w; 8 |
| 19 | Male | 57 | PD-1+TACE | -200 | -300 | / | Pembrolizumab | 200mg | q3w; 5 |
| 20 | Male | 53 | PD-1+TACE | -80 | -100 | / | Pembrolizumab | 200mg | q3w; 2 |
| 21 | Male | 54 | PD-1+TACE | -78 | -106 | / | Pembrolizumab | 200mg | q3w; 2 |
| 22 | Male | 68 | PD-1+TACE | -266 | -629 | / | Sintilimab | 200mg | q3w; 7 |
| 23 | Male | 72 | PD-1+TACE | -251 | -300 | / | Sintilimab | 200mg | q3w; 4 |
| 24 | Male | 58 | PD-1+TACE | -64 | -67 | / | Sintilimab | 200mg | q3w; 2 |
| 25 | Male | 58 | PD-1+TACE+TKI | -256 | -385 | -256 | Nivolumab | 200mg | q3w; 6 |
| 26 | Male | 66 | PD-1+TACE+TKI | -105 | -139 | -105 | Sintilimab | 200mg | q3w; 4 |
| 27 | Male | 49 | PD-1+TACE+TKI | -127 | -196 | -127 | Sintilimab | 200mg | q3w; 5 |
| 28 | Male | 63 | PD-1+TACE+TKI | -253 | -374 | -253 | Sintilimab | 200mg | q3w; 8 |
| 29 | Male | 62 | PD-1+TACE+TKI | -45 | -53 | -45 | Toripalimab | 480mg | 1 |
| 30 | Male | 51 | PD-1+TACE+TKI | -139 | -168 | -139 | Sintilimab | 200mg | q3w; 5 |
| 31 | Male | 50 | PD-1+TACE+TKI | -405 | -440 | -405 | Sintilimab | 200mg | q3w; 10 |
| 32 | Male | 40 | PD-1+TACE+TKI | -291 | -320 | -291 | Pembrolizumab | 200mg | q3w; 8 |
| 33 | Female | 60 | PD-1+TACE+TKI | -105 | -108 | -105 | Sintilimab | 200mg | q3w; 4 |
| 34 | Male | 61 | PD-1+TACE+TKI | -74 | -80 | -74 | Camrelizumab | 200mg | q3w; 2 |
| 35 | Male | 42 | PD-1+TACE+TKI | -466 | -556 | -466 | Camrelizumab | 200mg | q3w; 5 |
| 36 | Male | 56 | PD-1+TACE+TKI | -176 | -206 | -176 | Camrelizumab | 200mg | q3w; 6 |
| 37 | Male | 44 | PD-1+TACE+TKI | -85 | -101 | -85 | Camrelizumab | 200mg | q3w; 2 |
| 38 | Male | 64 | PD-1+TACE+TKI | -90 | -133 | -90 | Camrelizumab | 200mg | q3w; 2 |
| 39 | Female | 68 | PD-1+TACE+TKI | -95 | -106 | -95 | Camrelizumab | 200mg | q3w; 2 |
| 40 | Male | 50 | PD-1+TKI | -157 | / | -157 | Sintilimab | 200mg | q3w; 3 |
| 41 | Male | 45 | PD-1+TKI | -251 | / | -251 | Sintilimab | 200mg | q3w; 10 |
| 42 | Male | 57 | PD-1+TKI | -99 | / | -99 | Pembrolizumab | 200mg | q3w; 3 |
| 43 | Male | 36 | PD-1+TKI | -61 | / | -61 | Pembrolizumab | 200mg | q3w; 2 |
| 44 | Male | 64 | PD-1+TKI | -51 | / | -51 | Camrelizumab | 200mg | q3w; 2 |
| 45 | Male | 58 | PD-1+TKI | -158 | / | -158 | Camrelizumab | 200mg | q3w; 6 |
| 46 | Male | 59 | PD-1+TKI | -77 | / | -77 | Camrelizumab | 200mg | q3w; 2 |
| 47 | Male | 35 | PD-1+TKI | -74 | / | -74 | Toripalimab | 200mg | q3w; 2 |
| 48 | Male | 56 | PD-1+TKI | -28 | / | -28 | Toripalimab | 480mg | 1 |
| 49 | Male | 52 | PD-1+TKI | -27 | / | -27 | Toripalimab | 480mg | 1 |
| 50 | Male | 66 | PD-1+TKI | -75 | / | -75 | Camrelizumab | 200mg | q2w; 3 |
| 51 | Female | 54 | PD-1+TKI | -81 | / | -81 | Camrelizumab | 200mg | q3w; 2 |
| 52 | Male | 49 | PD-1+TKI | -27 | / | -27 | Toripalimab | 480mg | 1 |
| 53 | Male | 51 | PD-1+TKI | -25 | / | -25 | Toripalimab | 480mg | 1 |
| 54 | Male | 72 | PD-1+TKI | -23 | / | -23 | Toripalimab | 480mg | 1 |
| 55 | Male | 36 | PD-1+TKI | -30 | / | -30 | Toripalimab | 480mg | 1 |
| 56 | Male | 51 | PD-1+TKI | -73 | / | -73 | Camrelizumab | 200mg | q2w; 3 |
| 57 | Male | 50 | PD-1+TKI | -112 | / | -112 | Sintilimab | 200mg | q3w; 4 |
| 58 | Male | 57 | PD-1+TKI | -43 | / | -43 | Camrelizumab | 200mg | q2w; 2 |
| 59 | Male | 40 | PD-1+TKI | -41 | / | -41 | Camrelizumab | 200mg | q2w; 2 |
| 60 | Male | 54 | PD-1+TKI | -41 | / | -41 | Camrelizumab | 200mg | q2w; 2 |
| 61 | Male | 57 | PD-1+TKI | -41 | / | -41 | Camrelizumab | 200mg | q2w; 2 |
| 62 | Male | 58 | PD-1+TKI | -36 | / | -36 | Camrelizumab | 200mg | q2w; 2 |
| 63 | Male | 68 | PD-1+TKI | -36 | / | -36 | Camrelizumab | 200mg | q2w; 2 |
| 64 | Male | 55 | PD-1+TKI | -35 | / | -35 | Camrelizumab | 200mg | q2w; 2 |
| 65 | Male | 74 | PD-1+TKI | -38 | / | -38 | Camrelizumab | 200mg | q2w; 2 |
| 66 | Male | 66 | PD-1+TKI | -41 | / | -41 | Camrelizumab | 200mg | q2w; 2 |
| 67 | Male | 52 | PD-1+TKI | -36 | / | -36 | Camrelizumab | 200mg | q2w; 2 |
| 68 | Male | 51 | PD-1+TKI | -35 | / | -35 | Camrelizumab | 200mg | q2w; 2 |
| 69 | Male | 50 | PD-1+TKI | -35 | / | -35 | Camrelizumab | 200mg | q2w; 2 |
| 70 | Male | 51 | PD-1+TKI | -34 | / | -34 | Camrelizumab | 200mg | q2w; 2 |
| **Table Notes:** | | |  |  |  |  |  |  |  |
| **PD-1/TACE/TKI Relative Time (days):** Calculated relative to the surgery date (defined as day 0). Negative values indicate the number of days before surgery. | | | | | | | | | |
| **Concurrent:** In the TKI Time column, indicates that TKI therapy was initiated at the same time as PD-1 therapy. | | | | | | | | |  |
| **Cycle Abbreviations:** q3w (every 3 weeks), q2w (every 2 weeks). | | | | | |  |  |  |  |
