# Supplementary material for: An early relapse prediction model based on pathological features following neoadjuvant immunotherapy for hepatocellular carcinoma
Source: Oncologist. 2025 Nov 10;31(1):oyaf368. doi: 10.1093/oncolo/oyaf368 (PMC12771520; doi:10.1093/oncolo/oyaf368)
Supplement: oyaf368_Supplementary_Data [file oyaf368_supplementary_data.zip › Supplemental Material 2.docx]

| **Supplemental Material 2: The density of immune markers in different regions** | | | | | | | | | | | |
| --- | --- | --- | --- | --- | --- | --- | --- | --- | --- | --- | --- |
| marker/region（/mm2） | CD3/CT | CD3/NL | CD3/IM | CD4/CT | CD4/NL | CD4/IM | CD8/CT | CD8/NL | CD8/IM | CD15/CT | CD15/NL |
| 1 | 64.84 | 98.17 | 39.04 | 11.19 | 3.65 | 23.86 | 33.56 | 85.84 | 173.06 | 13.47 | 41.10 |
| 2 | - | 66.21 | - | - | 9.36 | - | - | 102.74 | - | - | 14.16 |
| 3 | 183.11 | 86.53 | 158.56 | 9.59 | 7.99 | 32.08 | 99.77 | 172.15 | 140.98 | 3.20 | 46.80 |
| 4 | - | - | - | - | - | - | - | - | - | - | - |
| 5 | 357.53 | 123.29 | 358.45 | 99.54 | 22.15 | 105.94 | 68.04 | 113.93 | 165.53 | 18.04 | 56.85 |
| 6 | 1097.26 | 342.92 | 1143.38 | 192.92 | 146.12 | 120.89 | 628.77 | 209.13 | 773.06 | 36.07 | 69.63 |
| 7 | 175.57 | 109.59 | 194.52 | 44.98 | 6.39 | 29.00 | 88.13 | 106.62 | 152.51 | 106.39 | 41.32 |
| 8 | - | - | - | - | - | - | - | - | - | - | - |
| 9 | 12.10 | 123.74 | 176.14 | 12.33 | 29.22 | 35.62 | 11.64 | 97.49 | 135.16 | 20.09 | 85.84 |
| 10 | 616.44 | 1047.26 | 86.76 | 305.48 | 715.07 | 34.02 | 699.54 | 1022.15 | 119.86 | 136.99 | 34.25 |
| 11 | 970.55 | 445.89 | 1255.71 | 50.23 | 12.10 | 177.74 | 777.17 | 1155.48 | 1098.86 | 48.86 | 51.37 |
| 12 | 168.04 | 224.89 | 362.79 | 20.78 | 12.10 | 29.79 | 69.41 | 149.32 | 287.90 | 18.26 | 31.05 |
| 13 | - | 87.55 | - | - | 20.33 | - | - | 89.74 | - | - | 13.01 |
| 14 | - | - | - | - | - | - | - | - | - | - | - |
| 15 | - | - | - | - | - | - | - | - | - | - | - |
| 16 | 393.15 | 79.68 | 360.50 | 25.11 | 18.95 | 30.71 | 174.89 | 107.76 | 260.16 | 427.40 | 91.78 |
| 17 | - | 40.87 | - | - | 41.10 | - | - | 91.32 | - | - | 4.57 |
| 18 | 81.28 | 99.09 | 155.82 | 13.47 | 8.22 | 39.04 | 55.02 | 98.40 | 145.32 |  |  |
| 19 | 221.23 | 43.15 | 147.95 | 8.90 | 7.08 | 21.80 | 70.78 | 40.64 | 138.47 | 48.40 | 96.80 |
| 20 | 378.61 | 154.11 | 359.59 | 296.04 | 58.60 | 140.41 | 176.56 | 103.50 | 140.41 | 54.79 | 62.79 |
| 21 | - | 53.42 | - | - | 17.58 | - | - | 109.13 | - | - | 18.72 |
| 22 | - | - | - | - | - | - | - | - | - | - | - |
| 23 | - | 547.95 | - | - | 136.99 | - | - | 399.54 | - | - | 57.08 |
| 24 | - | 47.72 | - | - | 10.73 | - | - | 38.13 | - | - | 28.54 |
| 25 | 21.23 | 19.86 | 130.71 | 8.45 | 4.34 | 15.98 | 44.52 | 69.86 | 150.68 | 19.63 | 75.34 |
| 26 | 228.08 | 261.64 | 124.54 | 89.50 | 146.35 | 40.07 | 54.41 | 262.94 | 54.79 | 33.11 | 32.72 |
| 27 | 130.14 | 399.54 | 242.96 | 18.65 | 16.74 | 12.56 |  |  |  | 15.22 | 114.92 |
| 28 | - | 76.94 | - | - | 63.93 | - | - | 74.20 | - | - | 27.17 |
| 29 | 375.95 | 168.95 | 351.03 | 15.98 | 23.21 | 26.07 | 45.66 | 51.37 | 97.41 | 44.52 | 65.45 |
| 30 | - | 31.96 | - | - | 18.26 | - | - | 45.66 | - | - | 11.64 |
| 31 | - | - | - | - | - | - | - | - | - | - | - |
| 32 | 17.58 | 40.87 | 35.27 | 9.59 | 31.96 | 9.59 | 12.79 | 14.38 | 39.04 | 2.05 | 16.44 |
| 33 | 182.65 | 22.83 | 114.16 | 23.97 | 14.61 | 25.11 | 93.61 | 45.66 | 127.85 | 12.56 | 10.96 |
| 34 | - | 19.41 | - | - | 7.76 | - | - | 15.75 | - | - | 20.09 |
| 35 | 191.10 | 144.75 | 239.27 | 26.03 | 18.26 | 15.87 | 84.93 | 98.63 | 199.32 | 37.90 | 151.60 |
| 36 | 241.25 | 108.83 | 283.30 | 183.03 | 52.13 | 156.01 | 143.84 | 154.49 | 286.53 | 32.34 | 182.65 |
| 37 | 530.14 | 490.87 | 603.20 | 22.83 | 46.12 | 46.80 | 528.08 | 521.69 | 659.02 | 104.11 | 87.90 |
| 38 | - | 61.64 | - | - | 18.26 | - | - | 65.53 | - | - | 14.16 |
| 39 | - | 38.81 | - | - | 76.03 | - | - | 38.58 | - | - | 22.83 |
| 40 | 170.32 | 227.63 | 457.19 | 57.53 | 10.73 | 101.83 | 108.45 | 209.36 | 445.32 | 49.32 | 92.47 |
| 41 | 663.70 | 72.15 | 825.11 | 163.24 | 8.45 | 189.04 | 656.62 | 65.07 | 743.95 | 226.48 | 33.56 |
| 42 | 148.40 | 229.22 | 507.65 | 30.82 | 60.05 | 43.72 | 99.54 | 284.25 | 191.78 | 261.64 | 83.79 |
| 43 | 329.68 | 144.98 | 257.42 | 74.43 | 15.98 | 58.11 | 178.77 | 138.81 | 229.79 | 15.98 | 52.51 |
| 44 | - | 28.54 | - | - | 20.32 | - | - | 58.68 | - | - | 12.79 |
| 45 | 239.95 | 540.87 | 511.07 | 13.93 | 36.76 | 128.77 | 214.61 | 256.39 | 374.09 | 29.00 | 102.05 |
| 46 | - | - | - | - | - | - | - | - | - | - | - |
| 47 | 378.54 | 208.68 | 411.87 | 86.99 | 46.12 | 74.20 | 376.26 | 184.93 | 383.45 | 9.36 | 74.66 |
| 48 | 152.51 | 221.92 | 446.23 | 26.94 | 6.85 | 136.07 | 114.61 | 165.98 | 330.94 | 41.10 | 48.40 |
| 49 | 146.80 | 86.53 | 477.74 | 10.27 | 3.88 | 51.48 | 185.84 | 95.43 | 559.93 | 13.93 | 45.21 |
| 50 | 501.14 | 284.47 | 344.63 | 24.89 | 7.31 | 2.28 | 390.18 | 273.06 | 230.82 | 93.38 | 43.38 |
| 51 | 1111.42 | 200.46 | 897.15 | 18.26 | 24.89 | 196.69 | 1266.21 | 218.49 | 670.89 | 33.11 | 25.34 |
| 52 | 144.75 | 138.58 | 183.68 | 6.85 | 12.56 | 9.25 | 134.93 | 124.43 | 152.74 | 10.73 | 79.45 |
| 53 | 145.19 | 78.34 | 156.78 | 18.14 | 23.12 | 4.56 | 123.34 | 99.15 | 78.16 | 50.68 | 46.35 |
| 54 | 632.88 | 131.05 | 369.06 | 75.57 | 15.30 | 79.79 | 341.55 | 112.33 | 169.52 | 136.30 | 74.20 |
| 55 | 552.74 | 103.65 | 779.79 | 73.52 | 19.63 | 96.80 | 650.23 | 163.24 | 902.17 | 8.68 | 63.24 |
| 56 | 133.56 | 233.79 | 381.05 | 29.68 | 58.22 | 82.19 | 205.71 | 298.63 | 334.59 | 45.66 | 99.77 |
| 57 | - | 25.11 | - | - | 9.13 | - | - | 25.57 | - | - | 14.16 |
| 58 | 344.29 | 145.89 | 386.42 | 81.51 | 11.87 | 270.66 | 233.33 | 100.68 | 363.81 | 20.09 | 67.12 |
| 59 | 267.35 | 231.96 | 424.89 | 27.63 | 14.61 | 14.27 | 135.39 | 209.36 | 423.52 | 44.52 | 44.29 |
| 60 | 502.51 | 407.53 | 468.04 | 289.73 | 84.02 | 107.19 | 427.63 | 386.53 | 451.14 | 8.90 | 16.89 |
| 61 | 255.94 | 157.76 | 415.53 | 23.97 | 17.81 | 67.24 | 158.22 | 88.58 | 410.39 | 18.04 | 46.35 |
| 62 | 1134.02 | 251.37 | 803.54 | 356.85 | 47.49 | 294.06 | 960.05 | 175.57 | 487.56 | 4.34 | 51.37 |
| 63 | 570.32 | 356.85 | 976.83 | 34.70 | 27.63 | 52.85 | 467.58 | 266.44 | 671.58 | 18.04 | 40.41 |
| 64 | 139.73 | 136.76 | 356.39 | 31.96 | 48.17 | 153.42 | 42.69 | 153.65 | 296.92 | 16.89 | 37.67 |
| 65 | 850.23 | 173.06 | 457.31 | 71.46 | 27.85 | 45.32 | 381.51 | 107.76 | 246.92 | 76.94 | 45.66 |
| 66 | 373.74 | 299.09 | 437.67 | 163.47 | 22.15 | 152.97 | 146.35 | 206.85 | 348.97 | 57.76 | 48.40 |
| 67 | 1606.39 | 274.20 | 862.67 | 917.81 | 23.29 | 733.33 | 1052.51 | 167.81 | 665.18 | 9.13 | 19.86 |
| 68 | 121.92 | 51.83 | 249.89 | 29.00 | 5.94 | 74.54 | 96.12 | 95.43 | 190.53 | 28.77 | 15.53 |
| 69 | 65.53 | 75.11 | 293.04 | 25.11 | 12.33 | 94.29 | 45.21 | 75.34 | 155.48 | 23.29 | 121.46 |
| 70 | 394.06 | 265.07 | 545.09 | 99.54 | 24.20 | 144.98 | 368.04 | 218.04 | 367.58 | 23.97 | 56.85 |
| marker/region（/mm2） | CD15/IM | CD20/CT | CD20/NL | CD20/IM | CD38/CT | CD38/NL | CD38/IM | CD68/CT | CD68/NL | CD68/IM | CD206/CT |
| 1 | 26.26 | 7.08 | 44.06 | 108.33 | 9.13 | 31.05 | 40.07 | 86.30 | 167.35 | 73.06 | 7.31 |
| 2 | - | - | 19.86 | - | - | 23.74 | - | - | 54.11 | - | - |
| 3 | 23.74 | 18.04 | 22.15 | 60.73 | 10.73 | 18.95 | 16.55 | 225.57 | 193.15 | 202.85 | 11.42 |
| 4 | - | - | - | - | - | - | - | - | - | - | - |
| 5 | 57.65 | 60.27 | 19.18 | 76.94 | 24.66 | 45.21 | 70.89 | 192.24 | 73.52 | 133.90 | 47.72 |
| 6 | 56.28 | 327.85 | 128.08 | 324.32 | 384.70 | 274.66 | 558.22 | 633.11 | 283.79 | 310.96 | 217.35 |
| 7 | 40.18 | 15.75 | 18.72 | 30.71 | 4.57 | 10.73 | 7.42 | 117.12 | 75.80 | 67.81 | 9.36 |
| 8 | - | - | - | - | - | - | - | - | - | - | - |
| 9 | 57.08 | 0.23 | 12.33 | 31.96 | 1.37 | 38.13 | 9.82 | 35.16 | 71.69 | 75.91 | 16.21 |
| 10 | 52.51 | 36.53 | 189.50 | 30.82 | 114.16 | 502.51 | 236.30 | 68.49 | 125.57 | 78.77 | 11.42 |
| 11 | 36.42 | 122.83 | 138.36 | 294.52 | 614.84 | 442.24 | 640.75 | 143.15 | 149.77 | 73.29 | 90.64 |
| 12 | 23.52 | 63.93 | 10.27 | 116.32 | 4.34 | 28.08 | 43.49 | 62.79 | 74.20 | 90.18 | 6.39 |
| 13 | - | - | 38.13 | - | - | 15.98 | - | - | 74.32 | - | - |
| 14 | - | - | - | - | - | - | - | - | - | - | - |
| 15 | - | - | - | - | - | - | - | - | - | - | - |
| 16 | 326.48 | 13.93 | 8.68 | 41.67 | 61.64 | 43.38 | 50.23 | 202.51 | 206.85 | 224.43 | 41.32 |
| 17 | - | - | 27.40 | - | - | 19.41 | - | - | 50.23 | - | - |
| 18 |  | 25.57 | 5.71 | 45.55 | 33.56 | 15.07 | 25.91 | 205.02 | 106.85 | 171.00 | 158.90 |
| 19 | 57.88 | 5.02 | 10.50 | 26.83 | 4.57 | 8.68 | 34.59 | 59.59 | 85.16 | 57.76 | 15.98 |
| 20 | 42.24 | 163.62 | 104.64 | 234.21 | 45.28 | 70.40 | 51.37 | 233.26 | 369.10 | 252.85 | 7.23 |
| 21 | - | - | 17.58 | - | - | 18.72 | - | - | 93.84 | - | - |
| 22 | - | - | - | - | - | - | - | - | - | - | - |
| 23 | - | - | 684.93 | - | - | 45.66 | - | - | 114.16 | - | - |
| 24 | - | - | 5.71 | - | - | 10.05 | - | - | 106.62 | - | - |
| 25 | 103.08 | 0.68 | 9.59 | 10.84 | 14.84 | 22.83 | 15.98 | 118.95 | 258.68 | 148.06 | 12.10 |
| 26 | 28.54 | 27.40 | 157.15 | 33.68 | 24.73 | 66.21 | 29.87 | 147.64 | 135.08 | 82.38 | 80.67 |
| 27 | 38.81 | 8.37 | 42.62 | 48.90 | 72.30 | 20.17 | 51.18 | 292.24 | 100.08 | 173.33 | 21.31 |
| 28 | - | - | 23.52 | - | - | 56.85 | - | - | 127.85 | - | - |
| 29 | 37.86 | 24.35 | 9.13 | 69.44 | 40.72 | 16.74 | 32.91 | 257.99 | 235.92 | 184.17 | 16.74 |
| 30 | - | - | 13.01 | - | - | 9.36 | - | - | 57.08 | - | - |
| 31 | - | - | - | - | - | - | - | - | - | - | - |
| 32 | 7.65 | 1.37 | 5.71 | 8.11 | 2.28 | 0.91 | 7.31 | 34.25 | 58.45 | 37.21 | 17.12 |
| 33 | 7.99 | - | 11.42 | - | - | 11.42 | - | - | 79.91 | - | - |
| 34 | - | - | 5.02 | - | - | 7.53 | - | - | 93.38 | - | - |
| 35 | 113.81 | 0.46 | 13.70 | 21.12 | 5.02 | 17.58 | 45.21 | 199.54 | 193.84 | 221.69 | 39.95 |
| 36 | 129.38 | 39.19 | 7.99 | 64.12 | 14.84 | 23.59 | 29.30 | 160.20 | 160.96 | 172.37 | 42.24 |
| 37 | 66.21 | 41.55 | 56.16 | 59.47 | 68.72 | 192.92 | 166.32 | 186.30 | 164.61 | 158.45 | 6.62 |
| 38 | - | - | 36.30 | - | - | 14.84 | - | - | 136.99 | - | - |
| 39 | - | - | 10.96 | - | - | 15.98 | - | - | 154.57 | - | - |
| 40 | 75.80 | 12.79 | 38.58 | 177.17 | 6.16 | 29.68 | 74.32 | 117.58 | 341.32 | 102.97 | 12.56 |
| 41 | 195.66 | 46.12 | 10.05 | 292.92 | 117.58 | 36.30 | 159.36 | 219.86 | 310.05 | 257.99 | 224.20 |
| 42 | 64.50 | 29.68 | 44.75 | 100.23 | 3.65 | 100.23 | 116.55 | 264.38 | 192.69 | 103.77 | 76.48 |
| 43 | 34.13 | 103.88 | 22.15 | 80.82 | 36.76 | 26.94 | 64.04 | 172.37 | 187.44 | 119.18 | 23.97 |
| 44 | - | - | 15.07 | - | - | - | - | - | 125.57 | - | - |
| 45 | 73.40 | 6.39 | 58.45 | 134.70 | 19.63 | 72.15 | 88.01 | 199.09 | 644.29 | 359.36 | 10.27 |
| 46 | - | - | - | - | - | - | - | - | - | - | - |
| 47 | 23.40 | 105.25 | 18.95 | 134.36 | 13.93 | 78.08 | 78.31 | 171.46 | 181.28 | 154.34 | 84.25 |
| 48 | 28.65 | 29.45 | 28.31 | 121.46 | 8.45 | 44.75 | 119.06 | 260.96 | 348.86 | 214.27 | 55.25 |
| 49 | 27.97 | 8.22 | 17.81 | 111.19 | 47.26 | 24.43 | 262.33 | 160.96 | 167.35 | 208.45 | 27.17 |
| 50 | 10.05 | 65.07 | 11.64 | 39.16 | 31.05 | 31.96 | 35.05 | 206.85 | 132.65 | 182.08 | 22.60 |
| 51 | 29.22 | 190.87 | 68.26 | 279.91 | 15.75 | 49.54 | 100.23 | 112.79 | 207.76 | 153.65 | 36.53 |
| 52 | 54.68 | 5.71 | 9.82 | 23.17 | 9.82 | 19.63 | 19.86 | 184.02 | 208.90 | 171.35 | 40.18 |
| 53 | 30.48 | 20.78 | 12.79 | 50.46 | 2.97 | 40.41 | 28.88 | 176.99 | 112.13 | 160.80 | 0.46 |
| 54 | 117.92 | 133.56 | 20.32 | 49.32 | 70.09 | 20.09 | 16.78 | 165.07 | 234.02 | 264.95 | 25.57 |
| 55 | 40.75 | 61.64 | 21.00 | 177.40 | 26.71 | 17.12 | 20.89 | 100.23 | 112.56 | 80.25 | 22.83 |
| 56 | 122.37 | 90.87 | 124.66 | 208.33 | 31.28 | 36.99 | 41.78 | 83.11 | 297.72 | 200.46 | 12.79 |
| 57 | - | - | 13.24 | - | - | 17.58 | - | - | 43.15 | - | - |
| 58 | 66.55 | 163.24 | 11.42 | 236.07 | 31.96 | 23.97 | 33.68 | 294.98 | 229.00 | 295.21 | 16.67 |
| 59 | 47.37 | 31.74 | 15.75 | 72.83 | 20.55 | 11.42 | 90.98 | 277.85 | 290.64 | 347.83 | 120.09 |
| 60 | 12.79 | 55.48 | 29.45 | 28.65 | 16.89 | 35.39 | 74.32 | 238.36 | 270.55 | 177.05 | 7.76 |
| 61 | 43.61 | 23.52 | 15.30 | 76.94 | 5.71 | 24.20 | 29.91 | 208.45 | 270.09 | 283.22 | 6.16 |
| 62 | 19.63 | 24.66 | 23.74 | 79.45 | 50.00 | 73.06 | 96.80 | 253.65 | 140.64 | 191.44 | 58.22 |
| 63 | 39.61 | 63.93 | 37.21 | 267.58 | 23.74 | 21.00 | 79.45 | 202.51 | 203.42 | 254.79 | 14.61 |
| 64 | 28.42 | 14.38 | 51.83 | 133.90 | 7.53 | 21.69 | 20.55 | 134.47 | 159.36 | 157.53 | 10.05 |
| 65 | 16.32 | 7.53 | 6.62 | 29.00 | 10.27 | 3.42 | 32.19 | 174.20 | 361.19 | 332.53 | 29.45 |
| 66 | 47.72 | 53.20 | 15.98 | 71.46 | 24.89 | 40.64 | 20.55 | 103.20 | 168.26 | 217.69 | 7.76 |
| 67 | 7.76 | 368.49 | 14.38 | 358.56 | 184.47 | 23.97 | 115.87 | 338.58 | 135.39 | 308.68 | 632.88 |
| 68 | 19.52 | 55.94 | 10.05 | 25.57 | 3.42 | 6.62 | 19.41 | 144.29 | 197.26 | 38.70 | 10.73 |
| 69 | 92.47 | 8.45 | 13.70 | 52.28 | 4.79 | 21.46 | 38.01 | 258.22 | 376.26 | 236.19 | 43.15 |
| 70 | 75.46 | 58.22 | 13.01 | 104.00 | 46.35 | 30.14 | 92.58 | 256.16 | 335.39 | 222.03 | 43.15 |
| marker/region（/mm2） | CD206/NL | CD206/IM | FOXP3/CT | FOXP3/NL | FOXP3/IM | PD1/CT | PD1/NL | PD1/IM | PDL1/288 | PDL1/22C3 |  |
| 1 | 195.66 | 121.58 | 0.68 | 2.51 | 0 | 13.7 | 11.42 | 12.75 | 0 | 0.2 |  |
| 2 | 20.32 | - | - | 1.37 | - | 91.32 | 75.34 | 71.16 | - | - |  |
| 3 | 12.10 | 7.88 | 5.71 | 2.51 | 1.37 | 27.4 | 23.97 | 31.2 | 0 | 0.1 |  |
| 4 | - | - | - | - | - | - | - | - | - | - |  |
| 5 | 34.70 | 40.87 | 2.28 | 0.91 | 0.34 | 80.29 | 38.81 | 44.52 | 0.5 | 1 |  |
| 6 | 77.40 | 165.41 | 63.93 | 1.14 | 17.12 | 140.79 | 45.66 | 152.21 | 20 | 20 |  |
| 7 | 35.16 | 52.63 | 0.46 | 1.14 | 0.57 | 10.65 | 7.61 | 8.18 | 0 | 0 |  |
| 8 | - | - | - | - | - | - | - | - | - | - |  |
| 9 | 32.19 | 12.79 | 0.91 | 1.60 | 1.03 | 2.66 | 7.99 | 13.51 | 0 | 0 |  |
| 10 | 125.57 | 25.11 | 38.81 | 1.14 | 14.27 | 171.23 | 45.66 | 114.16 | 3 | 5 |  |
| 11 | 71.23 | 93.95 | 6.62 | 1.14 | 4 | 136.99 | 79.91 | 152.21 | 5 | 10 |  |
| 12 | 46.12 | 35.84 | 0.00 | 0.46 | 0 | 38.05 | 26.64 | 32.34 | 0.1 | 0.1 |  |
| 13 | 23.06 | - | - | 1.45 | - | - | 22.83 | - | - | - |  |
| 14 | - | - | - | - | - | - | - | - | - | - |  |
| 15 | - | - | - | - | - | - | - | - | - | - |  |
| 16 | 35.62 | 45.55 | 3.88 | 0.91 | 2.05 | 30.44 | 20.93 | 24.73 | 0.1 | 0.1 |  |
| 17 | 45.66 | - | - | 4.57 | - | - | - | 20.36 | - | - |  |
| 18 | 5.71 | 4.91 | 13.70 | 1.37 | 4 | 15.6 | 30.82 | 31.39 | 0.1 | 0.1 |  |
| 19 | 2.74 | 7.65 | 1.14 | 0.68 | 0 | 25.11 | 16.74 | 22.07 | 1 | 1 |  |
| 20 | 25.88 | 75.91 | 151.83 | 36.15 | 177.51 | 117.96 | 41.86 | 50.8 | 5 | 7 |  |
| 21 | 35.62 | - | - | 1.14 | - | 30.44 | 39.95 | 57.08 | - | - |  |
| 22 | - | - | - | - | - | - | - | - | - | - |  |
| 23 | 57.08 | - | - | 7.76 | - | - | 102.74 | - | - | - |  |
| 24 | 43.15 | - | - | 0.68 | - | - | 30.44 | - | - | - |  |
| 25 | 33.79 | 7.76 | 3.42 | 2.05 | 1.83 | 15.22 | 7.61 | 8.56 | 0 | 0 |  |
| 26 | 23.21 | 13.32 | 31.58 | 32.34 | 15.41 | 34.25 | 23.97 | 51.18 | 0 | 2 |  |
| 27 | 6.85 | 12.75 | 5.71 | 0.68 | 7.99 | 76.1 | 30.06 | 43.76 | 1 | 1 |  |
| 28 | 65.98 | - | - | 5.25 | - | - | 28.16 | - | - | - |  |
| 29 | 17.88 | 30.82 | 16.36 | 8.75 | 6.85 | 41.86 | 20.93 | 64.69 | 0 | 0 |  |
| 30 | 45.66 | - | - | 0.46 | - | - | 24.35 | - | - | - |  |
| 31 | - | - | - | - | - | - | - | - | - | - |  |
| 32 | 25.34 | 14.04 | 1.37 | 0.91 | 0.8 | 6.47 | 6.85 | 8.18 | 0 | 1 |  |
| 33 | 45.66 | 34.25 | - | 18.26 | - | 68.49 | 15.22 | 33.49 | - | - |  |
| 34 | 31.28 | - | - | 2.28 | - | 72.3 | 13.32 | 41.86 | - | - |  |
| 35 | 131.28 | 80.37 | 0.23 | 1.14 | 0 | 24.73 | 6.85 | 32.53 | 0 | 0 |  |
| 36 | 43.00 | 59.74 | 88.28 | 28.92 | 86 | 39.95 | 30.44 | 36.15 | 5 | 5 |  |
| 37 | 90.18 | 27.63 | 0.23 | 0.00 | 0 | 140.79 | 57.08 | 81.81 | 1 | 2 |  |
| 38 | 68.49 | - | - | 1.83 | - | - | 20.93 | - | - | - |  |
| 39 | 45.66 | - | - | 2.05 | - | 28.54 | 22.83 | 29.68 | - | - |  |
| 40 | 100.23 | 53.77 | 1.37 | 0.91 | 0.46 | 25.49 | 47.56 | 36.72 | 0.5 | 3 |  |
| 41 | 64.61 | 169.18 | 6.85 | 0.91 | 6.85 | 79.91 | 19.41 | 60.88 | 0 | 1 |  |
| 42 | 104.57 | 45.78 | 0.91 | 1.14 | 1.37 | 15.98 | 22.07 | 18.07 | 0.1 | 2 |  |
| 43 | 21.46 | 81.96 | 0.91 | 0.68 | 0.68 | 32.72 | 4.57 | 18.84 | 0 | 0 |  |
| 44 | 70.55 | - | - | 0.91 | - | 57.08 | 38.05 | 60.88 | - | - |  |
| 45 | 26.26 | 13.01 | 1.37 | 2.05 | 5.14 | 95.13 | 38.05 | 72.3 | 1 | 10 |  |
| 46 | - | - | - | - | - | - | - | - | - | - |  |
| 47 | 92.47 | 62.33 | 11.42 | 0.91 | 1.26 | 45.66 | 49.47 | 25.88 | 0.5 | 0.5 |  |
| 48 | 73.29 | 51.37 | 2.51 | 0.91 | 0 | 6.85 | 34.25 | 10.65 | 0 | 0 |  |
| 49 | 18.72 | 55.02 | 11.42 | 0.68 | 16.21 | 51.37 | 11.42 | 33.3 | 0.1 | 0.1 |  |
| 50 | 9.82 | 21.23 | 1.83 | 0.00 | 0 | 26.64 | 19.03 | 19.79 | 0.1 | 1 |  |
| 51 | 52.05 | 34.25 | 31.96 | 2.97 | 11.99 | 194.06 | 22.83 | 117.96 | 5 | 10 |  |
| 52 | 49.77 | 61.76 | 0.00 | 0.00 | 0 | 5.33 | 15.22 | 10.65 | 0 | 0 |  |
| 53 | 30.14 | 9.70 | 1.14 | 0.00 | 3.42 | 15.22 | 18.65 | 63.17 | 0.3 | 0.5 |  |
| 54 | 45.43 | 51.37 | 0.91 | 0.46 | 0 | 15.22 | 12.18 | 20.36 | 3 | 3 |  |
| 55 | 125.57 | 62.79 | 9.13 | 0.46 | 10.84 | 68.49 | 34.25 | 95.13 | 3 | 5 |  |
| 56 | 22.60 | 23.97 | 3.20 | 0.68 | 6.28 | 10.65 | 34.25 | 23.21 | 0.1 | 1 |  |
| 57 | 20.32 | - | - | 0.68 | - | 57.08 | 24.35 | 47.56 | - | - |  |
| 58 | 7.31 | 15.53 | 2.51 | 1.14 | 4.34 | 26.64 | 11.42 | 32.34 | 0.1 | 0.1 |  |
| 59 | 33.56 | 36.99 | 12.10 | 11.42 | 4 | 20.93 | 41.86 | 19.6 | 0.1 | 0.2 |  |
| 60 | 59.36 | 123.40 | 19.63 | 7.99 | 15.41 | 106.54 | 22.83 | 26.64 | 2 | 5 |  |
| 61 | 2.05 | 15.07 | 15.07 | 4.11 | 2.97 | 57.08 | 76.1 | 55.18 | 0.1 | 0.1 |  |
| 62 | 51.14 | 48.86 | 34.47 | 2.97 | 30.82 | 190.26 | 76.1 | 116.06 | 70 | 70 |  |
| 63 | 7.76 | 14.50 | 56.85 | 1.37 | 28.2 | 117.96 | 38.05 | 83.71 | 5 | 5 |  |
| 64 | 9.59 | 17.35 | 7.08 | 3.88 | 7.53 | 12.56 | 15.98 | 26.07 | 0 | 0 |  |
| 65 | 5.25 | 22.49 | 19.86 | 1.37 | 9.7 | 64.69 | 14.46 | 45.66 | 10 | 10 |  |
| 66 | 25.57 | 14.61 | 3.20 | 0.68 | 0 | 58.98 | 60.88 | 47.56 | 0 | 0 |  |
| 67 | 18.26 | 180.71 | 114.16 | 3.42 | 79.91 | 140.79 | 38.05 | 111.87 | 1 | 1 |  |
| 68 | 17.12 | 17.12 | 2.97 | 2.74 | 3.77 | 29.3 | 15.22 | 18.07 | 2 | 2 |  |
| 69 | 45.66 | 25.46 | 4.34 | 2.74 | 1.6 | 8.75 | 12.94 | 13.89 | 1 | 1 |  |
| 70 | 45.89 | 29.22 | 14.16 | 1.83 | 7.99 | 62.79 | 34.25 | 31.39 | 5 | 5 |  |
| **Table Notes:** |  |  |  |  |  |  |  |  |  |  |  |
| **1. A "-" indicates that the number of immune cells cannot be calculated due to the complete necrosis of the tumor.** | | | | | | | | | |  |  |
| **2. CT: central tumor, IM: invasive margin, NL: normal liver.** | | | | | | | | | |  |  |
