# Supplementary material for: An early relapse prediction model based on pathological features following neoadjuvant immunotherapy for hepatocellular carcinoma
Source: Oncologist. 2025 Nov 10;31(1):oyaf368. doi: 10.1093/oncolo/oyaf368 (PMC12771520; doi:10.1093/oncolo/oyaf368)
Supplement: oyaf368_Supplementary_Data [file oyaf368_supplementary_data.zip › Supplemental Table 1.docx]

| **Supplemental Table 1. Relationship between different neoadjuvant treatment groups and histopathological morphology** | | | | | | | | | | | | | |
| --- | --- | --- | --- | --- | --- | --- | --- | --- | --- | --- | --- | --- | --- |
|  | All patients  N=90(%) | P^a^  N=16(%) | P+TA^b^  N=8(%) | P value | P+TA^b^  N=8(%) | TA^c^  n=20(%) | P value | P+TK+TA^d^  N=15(%) | TA^c^  N=20(%) | P value | P+TK^e^  N=31(%) | P^a^  N=16(%) | P value |
| Necrosis |  |  |  | 0.121 |  |  | 0.246 |  |  | 0.889 |  |  | 0.700 |
| Yes | 72(80) | 12(75.0) | 8(100.0) |  | 8(100.0) | 17(85.0) |  | 13(86.7) | 17(85.0) |  | 22(71.0) | 12(75.0) |  |
| No | 18(20) | 4(25.0) | 0(0.0) |  | 0(0.0) | 3(15.0) |  | 2(13.3) | 3(15.0) |  | 9(29.0) | 4(25.0) |  |
| TILs^f^ |  |  |  | 0.126 |  |  | **0.003** |  |  | **0.014** |  |  | 0.609 |
| Yes | 46(51.1) | 9(56.3) | 7(87.5) |  | 7(87.5) | 5(25.0) |  | 10(66.7) | 5(25.0) |  | 15(48.4) | 9(56.3) |  |
| No | 44(48.9) | 7(43.7) | 1(12.5) |  | 1(12.5) | 15(75.0) |  | 5(33.3) | 15(75.0) |  | 16(51.6) | 7(43.8) |  |
| TLSs^g^ |  |  |  | 1.000 |  |  | 0.306 |  |  | 0.403 |  |  | 0.464 |
| Yes | 16(17.8) | 4(25.0) | 6(75.0) |  | 6(75.0) | 2 (10.0) |  | 3(20.0) | 2 (10.0) |  | 5(16.1) | 4(25.0) |  |
| No | 74(82.2) | 12(75.0) | 2(25.0) |  | 2(25.0) | 18(90.0) |  | 12(80.0) | 18(90.0) |  | 26(83.9) | 12(75.0) |  |
| Fibrillation |  |  |  | 0.699 |  |  | 0.159 |  |  | 0.207 |  |  | 0.195 |
| Yes | 73(81.1) | 13(81.3) | 7(87.5) |  | 7(87.5) | 12(60.0) |  | 12(80.0) | 12(60.0) |  | 29(93.5) | 13(81.3) |  |
| No | 17(18.9) | 3(18.7) | 1(12.5) |  | 1(12.5) | 8(40.0) |  | 3(20.0) | 8(40.0) |  | 2(6.5) | 3(18.8) |  |
| Bleeding |  |  |  | 1.000 |  |  | 0.533 |  |  | 0.698 |  |  | 0.952 |
| Yes | 20(22.2) | 4(25.0) | 2(25.0) |  | 2(25.0) | 3(15.0) |  | 3(20.0) | 3(15.0) |  | 8(25.8) | 4(25.0) |  |
| No | 70(77.8) | 12(75.0) | 6(75.0) |  | 6(75.0) | 17(85.0) |  | 12(80.0) | 17(85.0) |  | 23(74.2) | 12(75.0) |  |
| Foam cell |  |  |  | 0.143 |  |  | **0.004** |  |  | **0.036** |  |  | 0.125 |
| Yes | 35(38.9) | 5(31.3) | 5(62.5) |  | 5(62.5) | 2(10.0) |  | 6(40.0) | 2(10.0) |  | 17(54.8) | 5(31.3) |  |
| No | 55(61.1) | 11(68.7) | 3(37.5) |  | 3(37.5) | 18(90.0) |  | 9(60.0) | 18(90.0) |  | 14(45.2) | 11(68.8) |  |
| Cholesterol crystal |  |  |  | 0.317 |  |  | **0.026** |  |  | 0.383 |  |  | 0.377 |
| Yes | 12(13.3) | 3(18.7) | 3(37.5) |  | 3(37.5) | 1(5.0) |  | 2(13.3) | 1(5.0) |  | 3(9.7) | 3(18.8) |  |
| No | 78(86.7) | 13(81.3) | 5(62.5) |  | 5(62.5) | 19(95.0) |  | 13(86.7) | 19(95.0) |  | 28(90.3) | 13(81.3) |  |
| ^a^ PD-1 group. ^b^ PD-1+TACE group. ^c^ TACE group. ^d^ PD-1+TKI+TACE group. ^e^ PD-1+TKI group. ^f^ tumor infiltrating lymphocytes. ^j^ ertiary lymphoid structures. | | | | | | | | | | | | | |
