# Supplementary figures and images for: An early relapse prediction model based on pathological features following neoadjuvant immunotherapy for hepatocellular carcinoma
Source: Oncologist. 2025 Nov 10;31(1):oyaf368. doi: 10.1093/oncolo/oyaf368 (PMC12771520; doi:10.1093/oncolo/oyaf368)

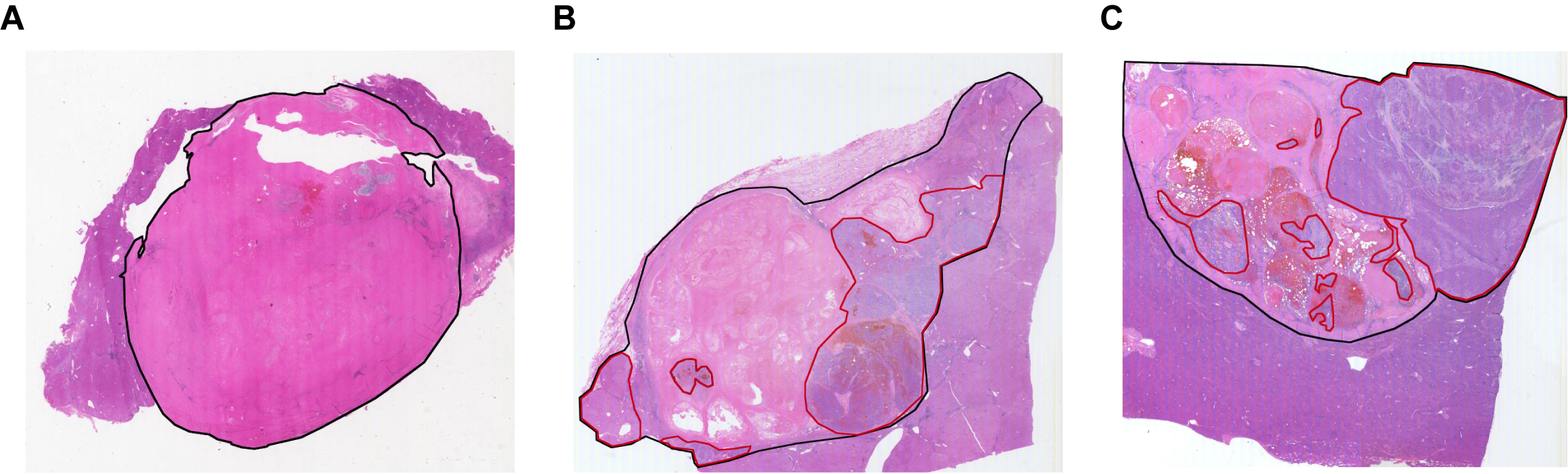

Supplement: oyaf368_Supplementary_Data [file oyaf368_supplementary_data.zip › Supplemental Figure 1.tif]

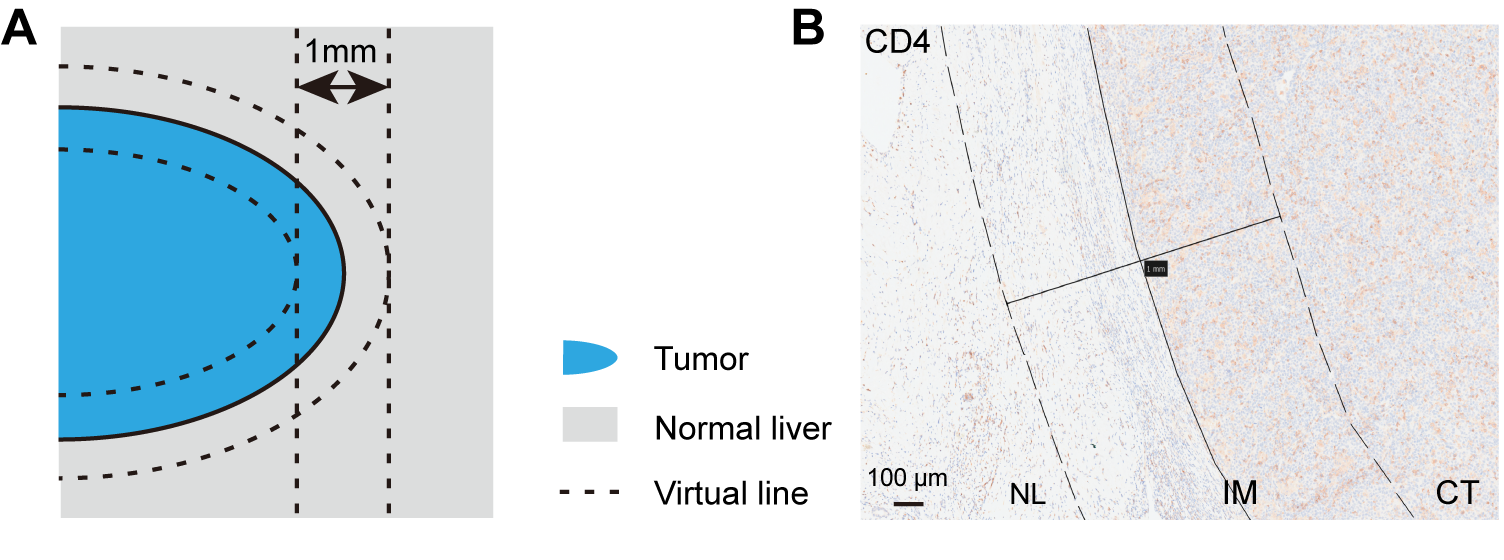

Supplement: oyaf368_Supplementary_Data [file oyaf368_supplementary_data.zip › Supplemental Figure 2.tif]
